# High‐Temperature Superconductivity in Perovskite Hydride Below 10 GPa

**DOI:** 10.1002/advs.202408370

**Published:** 2024-09-20

**Authors:** Mingyang Du, Hongyu Huang, Zihan Zhang, Min Wang, Hao Song, Defang Duan, Tian Cui

**Affiliations:** ^1^ Institute of High Pressure Physics School of Physical Science and Technology Ningbo University Ningbo 315211 P. R. China; ^2^ College of Physics Jilin University Changchun 130012 P. R. China

**Keywords:** first principles calculation, high pressure, hydrides, superconductivity

## Abstract

Hydrogen and hydride materials have long been considered promising materials for high‐temperature superconductivity. However, the extreme pressures required for the metallization of hydrogen‐based superconductors limit their applications. Here, a series of high‐temperature perovskite hydrides is designed that can be stable within 10 GPa. The research covered 182 ternary systems and ultimately determined that eight new compounds are stable within 20 GPa, of which five exhibited superconducting transition temperatures exceeding 120 K within 10 GPa, including KGaH_3_ (146 K at 10 GPa), RbInH_3_ (130 K at 6 GPa), CsInH_3_ (153 K at 9 GPa), RbTlH_3_ (170 K at 4 GPa) and CsTlH_3_ (163 K at 7 GPa). Excitingly, KGaH_3_ and RbGaH_3_ are thermodynamically stable at 50 GPa. Among these perovskite hydrides, alkali metals are responsible for providing a fixed amount of charge and supporting alloy framework composed of hydrogen and IIIA group elements to maintain stable crystal structure, while the cubic hydrogen alloy framework formed by IIIA group elements and hydrogen is crucial for high‐temperature superconductivity. This work will inspire further experimental exploration and take an important step in the exploration of low‐pressure stable high‐temperature superconductors.

## Graphical abstract

Perovskite hydrides AXH_3_ can achieve superconducting transition temperatures exceeding 150 K at pressure below 10 GPa, such as CsInH_3_ (153 K at 9 GPa), RbTlH_3_ (170 K at 4 GPa) and CsTlH_3_ (163 K at 7 GPa). In addition, KGaH_3_ (146 K at 10 GPa) and RbInH_3_ (130 K at 6 GPa) are thermodynamically stable at 50 GPa.

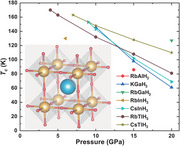

## 1 Introduction

Superconductors have unique physical properties such as complete conductivity and complete diamagnetism, which provide many possibilities for the development of human society. Superconductors have a wide variety of everyday applications, from MRI machines, maglev trains, particle accelerators, fusion reactors, to superconducting qubits in quantum computers. Researchers are now trying to find and develop superconductors that work at higher temperatures, which would revolutionize energy transport and storage. However, superconductivity can only typically be achieved at very cold temperatures. The discovery of superconductors with high transition temperatures (*T_c_*) has been a long‐standing hot topic in the scientific community, since the discovery of superconductivity of mercury in 1911.

According to the BCS theory,^[^ ^1^ ^]^ hydrogen, the lightest element with the highest Debye temperatures would be an ideal high‐temperature superconductor after metallization.^[^ ^2^ ^]^ However, hydrogen requires extremely high pressures to metallize.^[^ ^3^, ^4^ ^]^ Therefore, the search for high‐temperature superconductor has gradually turned to hydrogen‐rich compounds, as a more feasible route based on the ideas of “chemical pre‐compression”.^[^ ^5^ ^]^ In the past decade, many excellent hydrogen‐rich superconductors have been designed and predicted to be potential high‐temperature superconductors in the past decade,^[^ ^6^, ^7^, ^8^, ^9^, ^10^, ^11^, ^12^, ^13^ ^]^ some of which have been experimentally confirmed, including H_3_S,^[^ ^14^, ^15^ ^]^ LaH_10_,^[^ ^16^, ^17^ ^]^ ThH_9,10_,^[^ ^18^ ^]^ YH_9_,^[^ ^19^, ^20^ ^]^ CaH_6_,^[^ ^21^, ^22^ ^]^ YH_6_,^[^ ^23^ ^]^ CeH_9_,^[^ ^24^ ^]^ (La, Y)H_10_,^[^ ^25^ ^]^ (La, Ce)H_9,10_.^[^ ^26^, ^27^ ^]^

Hydride superconductors have shown great potential in high‐temperature superconductivity, but excessive pressure (> 100 GPa) still poses great difficulties for the experimental research and practical application of such superconducting materials. Therefore, achieving high‐temperature superconductivity under mild pressure conditions (< 50 GPa, the pressure limit of Kawai‐type multi‐anvil presses) is a key scientific problem that urgently needs to be solved in this field. The superconducting transition temperatures of HgBaCaCuO can reach 134 K at ambient pressure^[^ ^28^ ^]^ and 164 K at 30 GPa,^[^ ^29^ ^]^ respectively. However, at present, there is no quantitative theory that can predict the superconducting properties of such unconventional superconductors, seriously obstructing to further exploration for new unconventional superconductors. Recently, many hydrides have been predicted to be stable at low pressure with considerably high *T_c_*, such as CaBH_5_ (83 K at 1 GPa^[^ ^30^ ^]^), ThBeH_8_ (113 K at 7 GPa^[^ ^31^ ^]^), KB_2_H_8_ (146 K at 12 GPa^[^ ^32^ ^]^), LuN_4_H_11_ (100 K at 20 GPa^[^ ^33^ ^]^), BaBiH_8_ (94 K at 20 GPa^[^ ^34^ ^]^), MgN_2_H_8_ (105 K at 30 GPa^[^ ^35^ ^]^), LaTh_3_H_24_ (198 K at 50 GPa^[^ ^36^ ^]^). Among them, LaBeH_8_ has recently been experimentally synthesized, reaching a superconducting transition temperature of 110 K at 80 GPa.^[^ ^37^ ^]^

For a considerable period of time, the focus of searching for high‐temperature superconductors has been on hydrogen‐rich compounds, especially clathrate hydrides. Indeed, high hydrogen content can lead to a large H‐derived electronic density of states near the Fermi level, ultimately reaching high temperatures or even room‐temperature superconductivity. However, relying on the pressure to dissociate hydrogen molecules and stabilize the lattice, this structure formed through covalent bonds cannot be stable under near ambient pressure. The interstices in metal alloys can spontaneously capture a small amount of H atoms, resulting in few‐hydrogen alloys that can remain stable under near ambient pressure, and some may even exhibit high‐temperature superconductivity, such as Al_4_H (54 K^[^ ^38^ ^]^), MgHCu_3_ (40 K^[^ ^39^ ^]^), Mg_2_IrH_6_ (59‐134 K^[^ ^40^ ^]^), KInH_3_ (73 K^[^ ^41^ ^]^), ZnHAl_3_ (83 K^[^ ^42^ ^]^). The electron–phonon coupling between metals and hydrogen results in these few‐hydrogen alloys exhibiting surprisingly high *T_c_*, and exploring the superconducting properties and physical mechanisms of these few‐hydrogen alloys will be an important way to discover high‐temperature superconductors near ambient pressure.

In this work, we have designed a series of high‐temperature superconductors that can be stable within 10 GPa based on the perovskite structure of *Pm*‐3*m*‐KInH_3_.^[^ ^41^ ^]^ Our research covered 182 ternary systems and ultimately determined that eight compounds were stable within 20 GPa, of which five exhibited superconducting transition temperatures exceeding 120 K within 10 GPa. Excitingly, KGaH_3_ and RbGaH_3_ are thermodynamically stable at 50 GPa. Among these perovskite hydrides, alkali metals are responsible for providing a fixed amount of charge and maintaining structural stability, while the cubic framework formed by IIIA group elements and hydrogen is crucial for high‐temperature superconductivity. This work will inspire further experimental exploration and take an important step in the exploration of low‐pressure stable high‐temperature superconductors.

## 2 Results and Discussion

### 2.1 High‐Throughput Calculations

**Figure 1:**
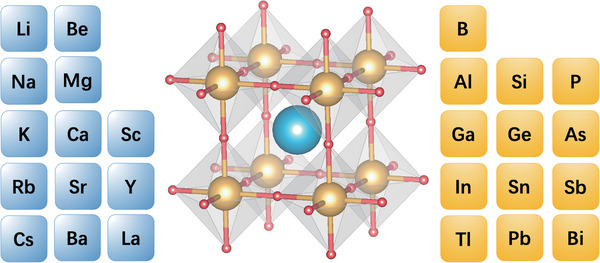
Crystal structure of the perovskite hydrides AXH_3_. Blue, yellow, and red balls represent A (group IA, IIA, or IIIB metal), X (a group IIIA, IVA, or VB element), and H atoms, respectively.

**Figure 2:**
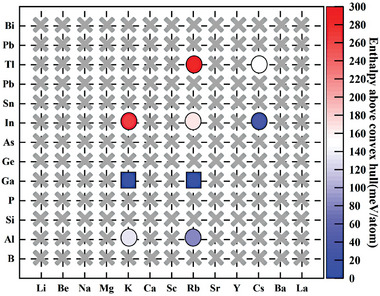
The circular marks indicate the dynamically stable perovskite hydrides AXH_3_ at 50 GPa. The square marks indicate the thermodynamically stable AXH_3_ at pressure lower than 50 GPa. The gray cross represents the AXH_3_ that are dynamically unstable at pressure lower than 50 GPa. The enthalpy values of AXH_3_ above the convex hull line are represented by the color of markers. The color bar indicates the correspondence between the color and values.

Using the perovskite structure of *Pm*‐3*m*‐KInH_3_ as a template, we obtained a series of AXH_3_ compounds by substituting the IA‐IVA and IIIB elements. The structure of AXH_3_ is shown in the middle of **Figure** **1**. The atom A selects Alkali metals, alkaline earth metals, and rare earth metals as shown at the left of Figure 1. The X selects IIIA, IVA, or VA elements as shown at the right of Figure 1. We first did a high‐throughput screening for the dynamical stability of perovskite hydrides AXH_3_ at 0–50 GPa. In this study, we found nine dynamically stable perovskite hydrides, KAlH_3_, RbAlH_3_, KGaH_3_, RbGaH_3_, KInH_3_, RbInH_3_, CsInH_3_, RbTlH_3_, CsTlH_3_, which are marked by circles in **Figure** **2**. The gray cross indicates that this structure cannot be stable within 50 GPa. Perhaps these structures can remain stable under higher pressures, but it is unlikely that these perovskite hydrides have *T_c_* exceeding 200 K, at pressures exceeding 50 GPa, they do not have significant advantages compared to other high‐temperature superconducting materials. Therefore, in this work, we will not study perovskite hydrides that can only be stable above 50 GPa. For materials, the mechanical stability can be obtained from elastic constants. We calculated their elastic constants using the strain–stress method. These results are listed in Table S2 (Supporting Information). According to our calculated results, all elastic constants meet the mechanical stability criteria which indicates their elastic stability.

For dynamically stable ternary perovskite hydrides, we further determined their thermodynamic stability (see Figures S1–S9, Supporting Information). All pertinent materials available in the materials project^[^ ^43^ ^]^ and previous works^[^ ^44^, ^45^ ^]^ were considered. Ab initio random structure searches were performed in each AXH_3_ compound at pressure of 0–50 GPa to supplement the enthalpy information of ternary compounds. The enthalpy values deviating from the ternary convex hull are shown as the color of markers in Figure 2. The bluer the color means more thermodynamically stable. The circular marks indicate that these structures are metastable within 50 GPa, and the square marks indicate that these structures are thermodynamically stable. In this study, we have uncovered two thermodynamically stable perovskite hydrides, KGaH_3_ and RbGaH_3_. In addition, it is worth mentioning that CsInH_3_ is only 38 meV per atom away from the convex hull. We constructed convex hull for CsInH_3_ every 10 GPa within the pressure range of 0–100 GPa. The enthalpy above convex hull of CsInH_3_ as a function of pressure is shown in Figure S13 (Supporting Information). It can be seen that at 20 GPa, CsInH_3_ is only 9 meV away from the convex hull. Which means that it still has the possibility of being synthesized experimentally. The other compounds, KAlH_3_ (138 meV per atom), RbAlH_3_ (83 meV per atom), RbInH_3_ (165 meV per atom), RbTlH_3_ (284 meV per atom), CsTlH_3_ (147 meV per atom), are too far from the convex hull. Even worse, we have found lower energy phases in the structure search, such as *Immm*‐KAlH_3_, *P*2_1_/*m*‐RbAlH_3_, *P*4/*mmm*‐RbInH_3_, *C*2/*m*‐RbTlH_3,_ and *P*‐1‐CsTlH_3_, which makes the synthesis of high‐*T_c_* phase for these compounds relatively difficult. For thermodynamically stable phase *Pm*‐3*m*‐KGaH_3_ and *Pm*‐3*m*‐RbGaH_3_, their enthalpy is the lowest and can be easily synthesized (see Figure S12b,d, Supporting Information). But for metastable phases such as *Pm*‐3*m*‐KAlH_3_, their synthesis is more difficult due to the presence of a lower enthalpy phase *Immm*‐KAlH_3_ (see Figure S12a, Supporting Information). It is highly possible to synthesize *Immm*‐KAlH_3_ instead of *Pm*‐3*m*‐KAlH_3_ during synthesis. The same situation can also occur on RbAlH_3_, RbInH_3_, RbTlH_3_ and CsTlH_3_. This will significantly increase the cost of experimental synthesis. The situation of the metastable phase *Pm*‐3*m*‐CsInH_3_ is much better because its synthesis only needs to avoid the occurrence of CsH_7_ and CsH_9_ (see Figure S12f, Supporting Information).

**Figure 3:**
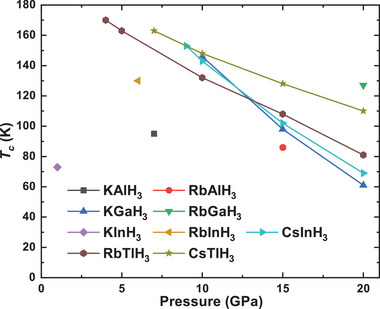
The *T_c_* s of perovskite hydrides AXH_3_ are estimated through the self‐consistent iteration solution of the Eliashberg equation as a function of pressures. The data of KInH_3_ was obtained from previous work.^[^ ^41^ ^]^

We calculated the electron‐phonon coupling (EPC) to estimate the superconductivity. The *T_c_* s are estimated through the self‐consistent iteration solution of the Eliashberg equation (scE) with µ^∗^ = 0.10 as a function of pressure are supplied in **Figure** **3**. RbTlH_3_ shows the highest *T_c_* of 170 K at 4 GPa, followed by CsTlH_3_ with *T_c_* of 163 K at 7 GPa. But unfortunately, both of them are metastable phases, which means that some difficulties need to be overcome in the experiment to synthesize these structures. Considering the continuous progress in the synthesis technology of metastable materials in experiments, there is also a considerable possibility that we will see these excellent properties of metastable materials successfully synthesized in the future.^[^ ^46^ ^]^ CsInH_3_ shows the highest *T_c_* of 153 K at 9 GPa in all thermodynamically stable perovskite hydrides, followed by KGaH_3_ with a *T_c_* of 146 K at 10 GPa. RbGaH_3_ requires more than 20 GPa to be dynamically stable, and its *T_c_* is expected to be 127 K at this time. In addition, the metastable phase RbInH_3_ also exhibits good properties, with a *T_c_* of 130 K at 6 GPa. Other metastable phase KAlH_3_ and RbAlH_3_ shows the *T_c_* of 95 K at 7 GPa and 86 K at 15 GPa respectively. Detailed computational results at different pressures are supplied in Table S3 in the Supporting Information. In addition, the *T_c_* s of these perovskite hydrides are more sensitive to pressure than other hydrides. RbTlH_3_ shows the *T_c_* of 170 K at 4 GPa, but as the pressure increased to 20 GPa, its *T_c_* rapidly decreased to 81 K. The *T_c_* of KGaH_3_, CsInH_3_, and CsTlH_3_ also rapidly decrease with increasing pressure. In previous calculations, the *T_c_* of KInH_3_ was 72.9 K at ambient pressure. And in our calculations, the *T_c_* of KInH_3_ in 71.6 K at ambient pressure, which is very close to previous results.

### 2.2 Charge‐Sensitive Hydrogen Alloy Framework

**Figure 4:**
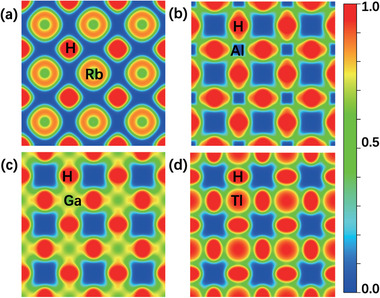
The 2‐D electron localization function (ELF) for perovskite hydrides (a) RbAlH_3_ at alkali metal layer, (b) RbAlH_3_, (c) RbGaH_3_ and (d) RbTlH_3_ at IIIA group atomic layer.

**Figure 5:**
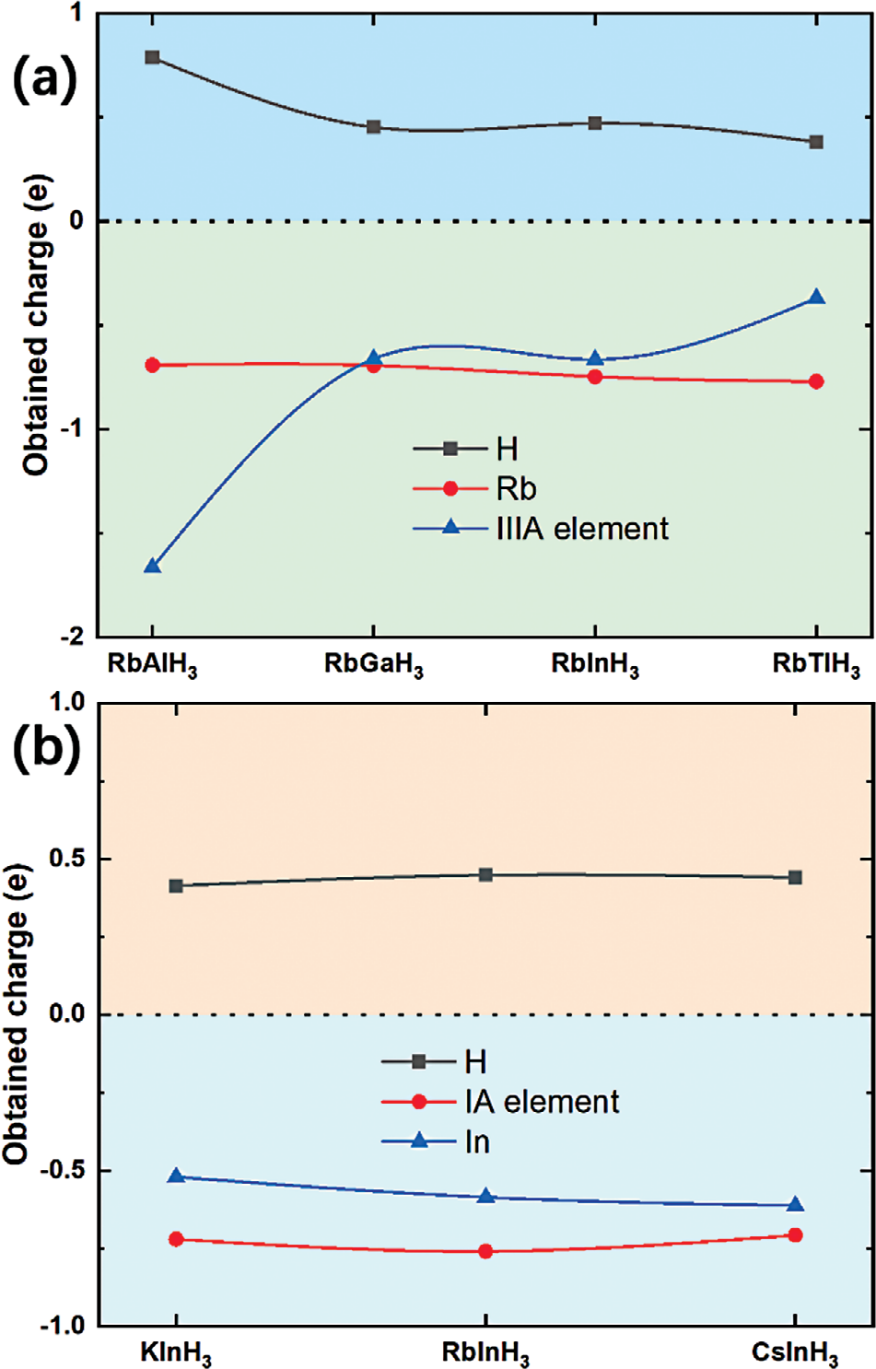
Remnant charges on A, X, and H atoms obtained from Bader Charge Analysis of (a) RbXH_3_ (X = Al, Ga, In, Tl) and (b) AInH_3_ (A = K, Rb, Cs).

We furthermore calculated the electron localization function (ELF) and Bader charge of these perovskite hydrides at different pressures (see **Figure** **4**). The structure of perovskite hydride AXH_3_ can be divided into two parts: alkali metals, and the framework structure formed by IIIA group elements and hydrogen. Figure 4a shows the ELF of the alkali metal layer of RbAlH_3_, charge localization around alkali metal Rb. This indicates that the alkali metal Rb plays a “pre‐compressor” role to support the framework structure formed by IIIA group elements and hydrogen in RbAlH_3_ and does not directly contribute to superconductivity. The ELF of the alkali metal layer in other AXH_3_ structures is basically consistent with Figure 4a (see Figure S15, Supporting Information), which means that alkali metals play a “pre‐compressor” role in all perovskite hydrides AXH_3_. From **Figure** **5**a, it can be seen that the electrons provided by the alkali metal Rb in RbXH_3_ (X = Al, Ga, In, Tl) remain in a relatively stable range (0.69 e–0.77 e). Even if replaced with other alkali metals with different radii, the electrons provided by alkali metals in AInH_3_ (A = K, Rb, Cs) remain within a relatively stable range (0.71 e–0.72 e). This means that alkali metals also act as electron donors in AXH_3_, and alkali metals provide a relatively fixed number of electrons. Changes in elements and pressure have little effect on the number of electrons provided by alkali metals.

Figure 4b–d shows the ELF of the IIIA group atomic layers in RbAlH_3_, RbGaH_3,_ and RbTlH_3_, respectively. The IIIA group atomic layers in different AXH_3_ exhibit significant differences, both in charge density and charge distribution. The Al layer in RbAlH_3_ has the lowest charge density in RbXH_3_ (X = Al, Ga, In, Tl), and the charges around H are distributed in an ellipsoidal shape, with the ellipsoidal axis along the Al‐H direction (see Figure 4b). The Ga layer in RbGaH_3_ has the highest charge density, and the charges around H are distributed in a spherical shape (see Figure 4c), and the ELF of the In layer in RbInH_3_ is also basically similar (see Figure S15, Supporting Information). The Tl layer in RbTlH_3_ has the moderate charge density, and the charges around H are distributed in an ellipsoidal shape, with the ellipsoidal axis perpendicular to the Tl‐H direction (see Figure 4d). It can be seen that the X‐H framework formed by IIIA group elements and hydrogen is the key to the superconductivity of the AXH_3_ system, and the charge density and distribution on the B‐H framework will affect the superconductivity of the system. From Figure 5b, it can also be seen that there are significant differences in charge transfer between different IIIA group elements, with Al transferring up to 1.66 e of charge, while Tl only transferring 0.37 e of charge. Considering the superconductivity and stability of each AXH_3_ in Figures 2 and 3, we believe that the Tl element with the least charge transfer (0.37 e) is more conducive to high‐temperature superconductivity and low‐pressure dynamic stability of the structure, while the Ga element with moderate charge transfer (0.66 e) are more conducive to thermodynamic stability of the structure.

**Figure 6:**
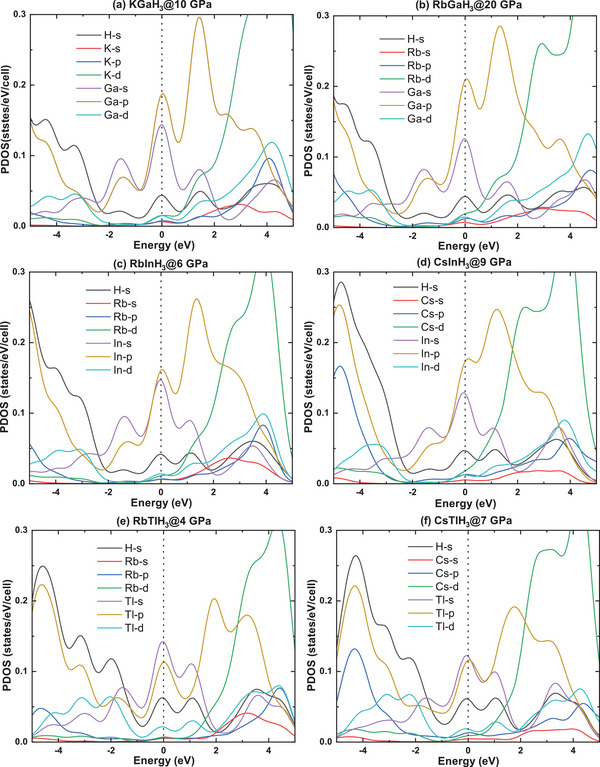
The projected electronic density of states of (a) KGaH_3_ at 10 GPa, (b) RbGaH_3_ at 20 GPa, (c) RbInH_3_ at 6 GPa, (d) CsInH_3_ at 9 GPa, (e) RbTlH_3_ at 4 GPa and (f) CsTlH_3_ at 7 GPa.

Subsequently, we calculated the electronic band structures (see Figure S16, Supporting Information) and projected density of states (PDOS) to understand the effect of pressure on the electronic structure for KGaH_3_ at 10 GPa, RbGaH_3_ at 20 GPa, RbInH_3_ at 3 GPa, CsInH_3_ at 9 GPa, RbTlH_3_ at 4 GPa and CsTlH_3_ at 7 GPa. As shown in **Figure** **6**, AXH_3_ share the similar electronic structure with peak of DOS located above the Fermi energy level, contributions from s‐orbitals and p‐orbitals in IIIA element dominate the DOS at the Fermi level. When the IIIA elements are the same, the electronic structure of AXH_3_ is basically the same, which is also consistent with the results in Figure 5b. The key difference in the electronic structure of AXH_3_ lies in the contribution of hydrogen on the Fermi surface. The contribution of H in the Fermi surface is higher in RbTlH_3_ and CsTlH_3_, which may be an important reason for their higher *T_c_*. Based on the charge transfer situation in Figure 5a, we believe that in AXH_3_, less charge transfer is more favorable for the DOS peak of hydrogen near the Fermi surface, leading to a higher superconducting transition temperature. The X‐H covalent framework is the key to AXH_3_ high‐temperature superconductivity. As hydrogen gains too many electrons and exhibits hydrogen ion properties, its DOS will appear more in deep energy levels rather than near the Fermi surface.

### 2.3 “Softening” Induced High‐Temperature Superconductivity

**Figure 7:**
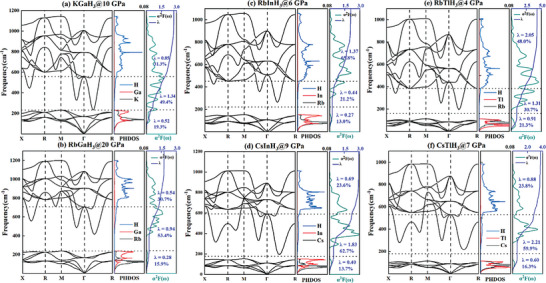
Calculated phonon dispersion curves, phonon density of states (PHDOS), Eliashberg spectral function α^2^F(ω) and the accumulated EPC constant λ of (a) KGaH_3_ at 10 GPa, (b) RbGaH_3_ at 20 GPa, (c) RbInH_3_ at 6 GPa, (d) CsInH_3_ at 9 GPa, (e) RbTlH_3_ at 4 GPa and (f) CsTlH_3_ at 7 GPa.

To further understand the source of high‐temperature superconductivity of these perovskite hydrides AXH_3_, we calculated the phonon band structure, projected phonon density of states (PHDOS), accumulated electron‐phonon coupling (EPC) parameter λ, and Eliashberg spectral function α^2^F(ω) of KGaH_3_ at 10 GPa, RbGaH_3_ at 20 GPa, RbInH_3_ at 3 GPa, CsInH_3_ at 9 GPa, RbTlH_3_ at 4 GPa and CsTlH_3_ at 7 GPa, as shown in **Figure** **7**. It can be seen from PHDOS that the low‐frequency phonon modes (below 200 cm^−1^) are mainly from IA and IIIA elements, high‐frequency phonon modes (above 200 cm^−1^) are mainly from H atom. The accumulated EPC constant λ in the low‐frequency region accounts for 13%–21.3% of the total λ, indicating that non‐hydrogen elements contribute less to the electron‐phonon coupling. The key of these perovskite hydrides AXH_3_ to high‐temperature superconductivity, like other hydrides, is also related to the high‐frequency vibration of hydrogen.

As we discussed in Figure 3, the *T_c_* s of these perovskite hydrides are more sensitive to pressure than other hydrides. From the phonon band structure, it is likely due to a significant contribution of phonon softening in the superconductivity of these structures. The accumulated EPC constant λ in the phonon softening region accounts for 21.2%–62.7% of the total λ, which is higher than the contribution of non‐hydrogen elements. Furthermore, it should be noted that a considerable portion of the electroacoustic coupling in the high‐frequency region also comes from phonon softening, such as phonon softening near the frequency of 600 cm^−1^ on the high symmetry path X‐R‐M in RbInH_3_ and RbTlH_3_ (see Figure 7c,e). And The growth of accumulated EPC constant λ is most rapid around the frequency ≈600 cm^−1^. Considering this, we can consider that phonon softening contributes more than 50% to the electron‐phonon coupling among the six perovskite hydrides AXH_3_ in Figure 7. When the pressure increases, phonon softening weakens, and the corresponding electron‐phonon coupling rapidly weakens (see Figure S17, Supporting Information), ultimately leading to the rapid decrease in *T_c_*. The calculated EPC constant λ, logarithmic average phonon frequency ω_log_, superconducting critical temperature *T_c_* for perovskite hydrides AXH_3_ at different pressures are listed in Table S3 in the Supporting Information. It can be clearly seen that as the pressure increases to 20 GPa, the λ of CsInH_3_ rapidly decreases from 2.92 to 1.24, and *T_c_* of CsInH_3_ decreases from 153 to 69 K; the λ of RbTlH_3_ decreases from 4.27 to 1.28, and *T_c_* of RbTlH_3_ decreases from 170 to 81 K; the λ of CsTlH_3_ decreases from 3.69 to 1.48, and *T_c_* of RbTlH_3_ decreases from 163 to 110 K. This high sensitivity of *T_c_* to pressure may result in higher requirements for precise pressure control in future experimental synthesis of such hydrides. But from another perspective, this sensitivity may allow them to be applied in more fields beyond high‐temperature superconductivity.

## 3 Conclusion 

In summary, we have designed a series of high‐temperature superconductors that can be stable within 10 GPa based on perovskite structure of *Pm*‐3*m*‐KInH_3_. Our research covered 182 ternary systems and ultimately determined that eight compounds were stable within 20 GPa, of which five exhibited superconducting transition temperatures exceeding 120 K within 10 GPa, including KGaH_3_ (146 K at 10 GPa), RbInH_3_ (130 K at 6 GPa), CsInH_3_ (153 K at 9 GPa), RbTlH_3_ (170 K at 4 GPa) and CsTlH_3_ (163 K at 7 GPa). Excitingly, KGaH_3_ and RbGaH_3_ are thermodynamically stable at 50 GPa. Among these perovskite hydrides, alkali metals are responsible for providing a fixed amount of charge and maintaining structural stability, while the cubic framework formed by IIIA group elements and hydrogen is crucial for high‐temperature superconductivity. EPC calculations revealed that the contribution of the softening phonon mode of hydrogen to the EPC constant λ exceeds 50%. This phonon softening enables these hydrides to exhibit unexpectedly high superconducting transition temperatures at low pressures, but as the pressure increases, the softening decreases, and their superconducting transition temperature rapidly decreases to less than half of its maximum *T_c_*. This high sensitivity of *T_c_* to pressure may result in higher requirements for precise pressure control in future experimental synthesis of such hydrides. But from another perspective, this sensitivity may allow them to be applied in more fields beyond high‐temperature superconductivity. This work will inspire further experimental exploration and take an important step in the exploration of low‐pressure stable high‐temperature superconductors.

## 4 Computational Methods

The crystal structure predictions of AXH_3_ compounds were carried out using Ab initio random structure searching (AIRSS) technique.^[^ ^47^, ^48^ ^]^ The candidate structures from the crystal structure prediction calculations were then used for subsequent DFT calculations using the Cambridge Serial Total Energy Package (CASTEP).^[^ ^49^ ^]^ Preliminary structural relaxation is performed using the Perdew–Burke–Ernzerhof (PBE) parametrization of the generalized gradient approximation (GGA)^[^ ^50^ ^]^ for the exchange‐correlation functional and on‐the‐fly generated ultrasoft pseudopotentials with plane‐wave cut off of 450 eV and k‐point spacing of 0.07 × 2π Å for structure searching. To generate the convex hull, high‐accuracy geometry optimizations of all structures were performed by using AIRSS, with plane‐wave cut off of 800 eV and k‐point spacing of 0.03 × 2π Å. All pertinent materials available in the materials project^[^ ^43^ ^]^ were considered and were re‐optimized.

The calculations of electronic properties, including electron localization function and Bader charge, were calculated by using the Vienna ab initio simulation program (VASP).^[^ ^51^ ^]^ The projector augmented plane‐wave (PAW) pseudopotentials were used with the generalized gradient approximation (GGA)^[^ ^50^ ^]^ for the exchange‐correlation functional and plane‐wave cut off of 800 eV. Brillouin zone (BZ) sampling using Monkhorst‐Pack^[^ ^52^ ^]^ meshes with k‐point spacing of 0.03 × 2π Å to ensure that all enthalpy calculations were well converged to less than 1 meV per atom (see Figure S18, Supporting Information).

The Quantum‐ESPRESSO code^[^ ^53^ ^]^ was used in electron–phonon calculations, including the phonon band structure, projected phonon density of states (PHDOS), and Eliashberg spectral function α^2^F(ω). The k‐points and q‐points meshes for the high‐throughput calculations were 12 × 12 × 12 and 3 × 3 × 3, respectively. All calculations were done using projector augmented plane‐wave (PAW) Perdew–Burke–Ernzerhof (PBE) pseudopotentials from PS Library and a 90 Ry energy cut off for the plane wave expansion to ensure that all enthalpy calculations are well converged (see Figure S19, Supporting Information). For the stable structures identified through high‐throughput screening, we selected denser k‐point and q‐point grids for higher accuracy calculations. The q‐points meshes for the final electron–phonon calculations were 6 × 6 × 6, respectively. The superconducting transition temperatures of these structures are estimated through the self‐consistent iteration solution of the Eliashberg equation (scE).^[^ ^54^ ^]^

## Conflict of Interest

The authors declare no conflict of interest.

## Supporting information



Supporting Information

## Data Availability

The data that support the findings of this study are available from the corresponding author upon reasonable request.
