# Supplementary material for: High‐Temperature Superconductivity in Perovskite Hydride Below 10 GPa
Source: Adv Sci (Weinh). 2024 Sep 20;11(42):2408370. doi: 10.1002/advs.202408370 (PMC11558092; doi:10.1002/advs.202408370)
Supplement: Supplementary file 1 — Supporting Information [file ADVS-11-2408370-s001.docx]

Supporting Information

High-temperature Superconductivity in Perovskite Hydride below 10 GPa

Mingyang Du, Hongyu Huang, Zihan Zhang, Min Wang, Hao Song*, Defang Duan, Tian Cui*


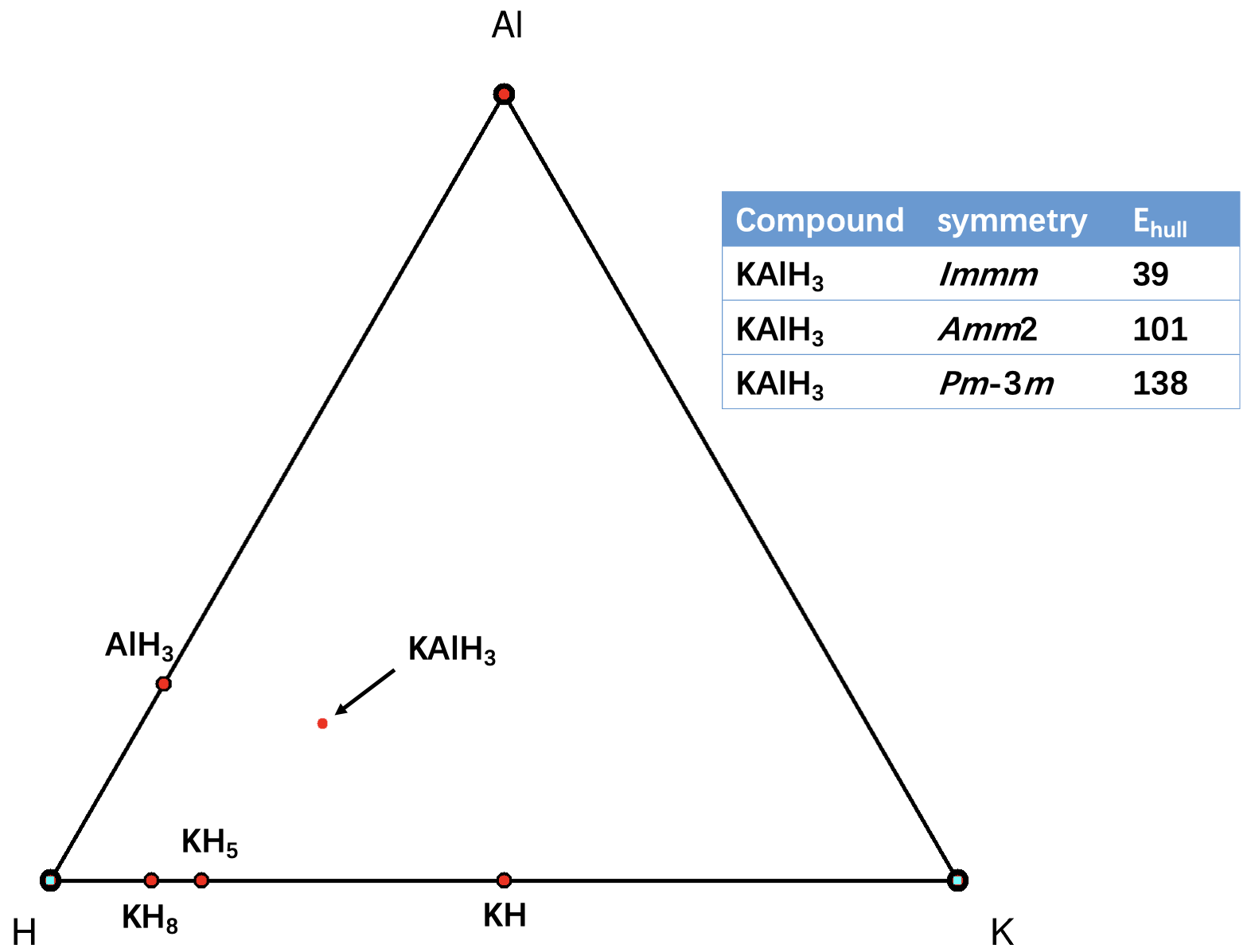


Figure S1 The convex hull of KAlH_3_ at 50 GPa. E_hull_ represent the distance to the convex hull of stability in meV per atom.


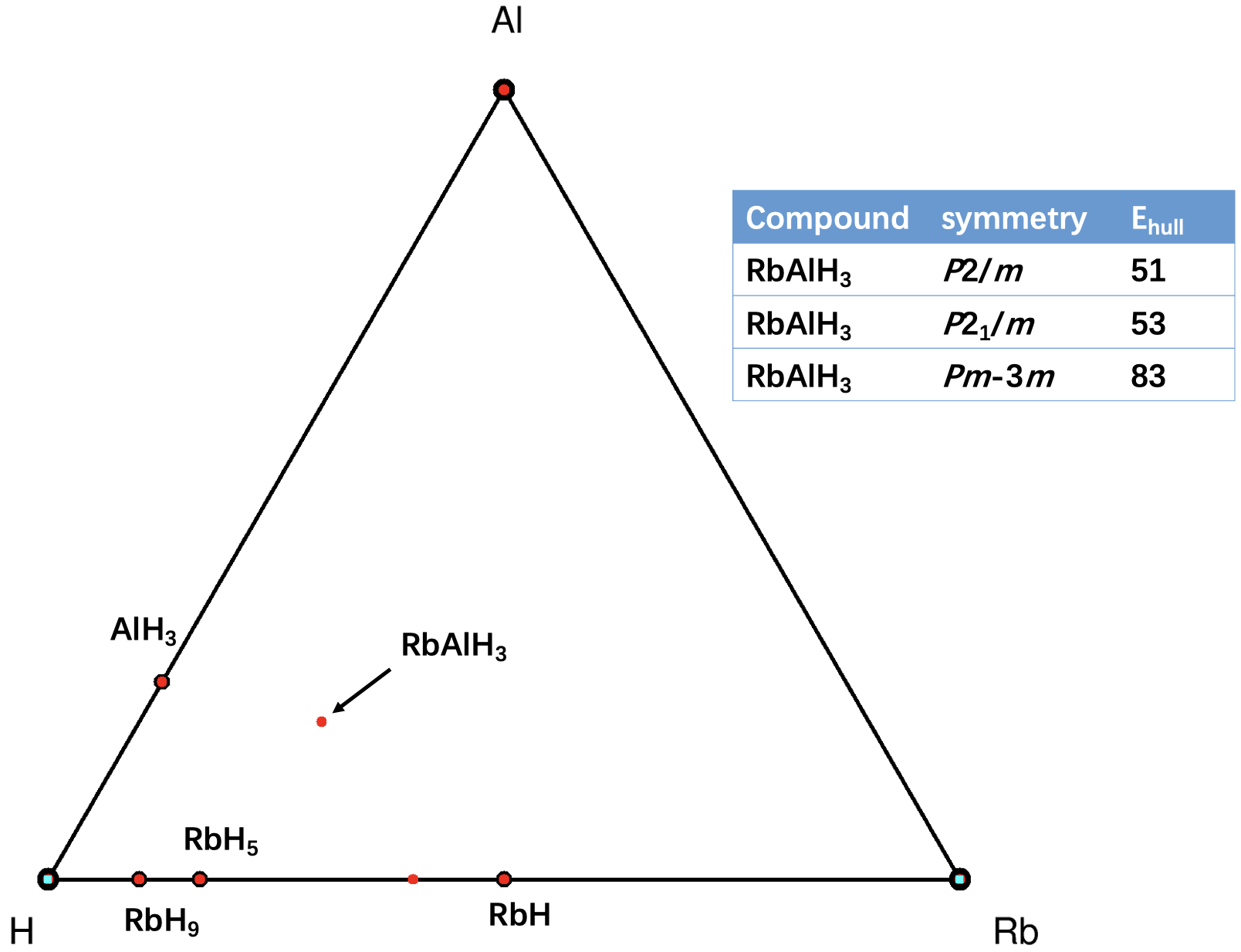


Figure S2 The convex hull of RbAlH_3_ at 50 GPa. E_hull_ represent the distance to the convex hull of stability in meV per atom.


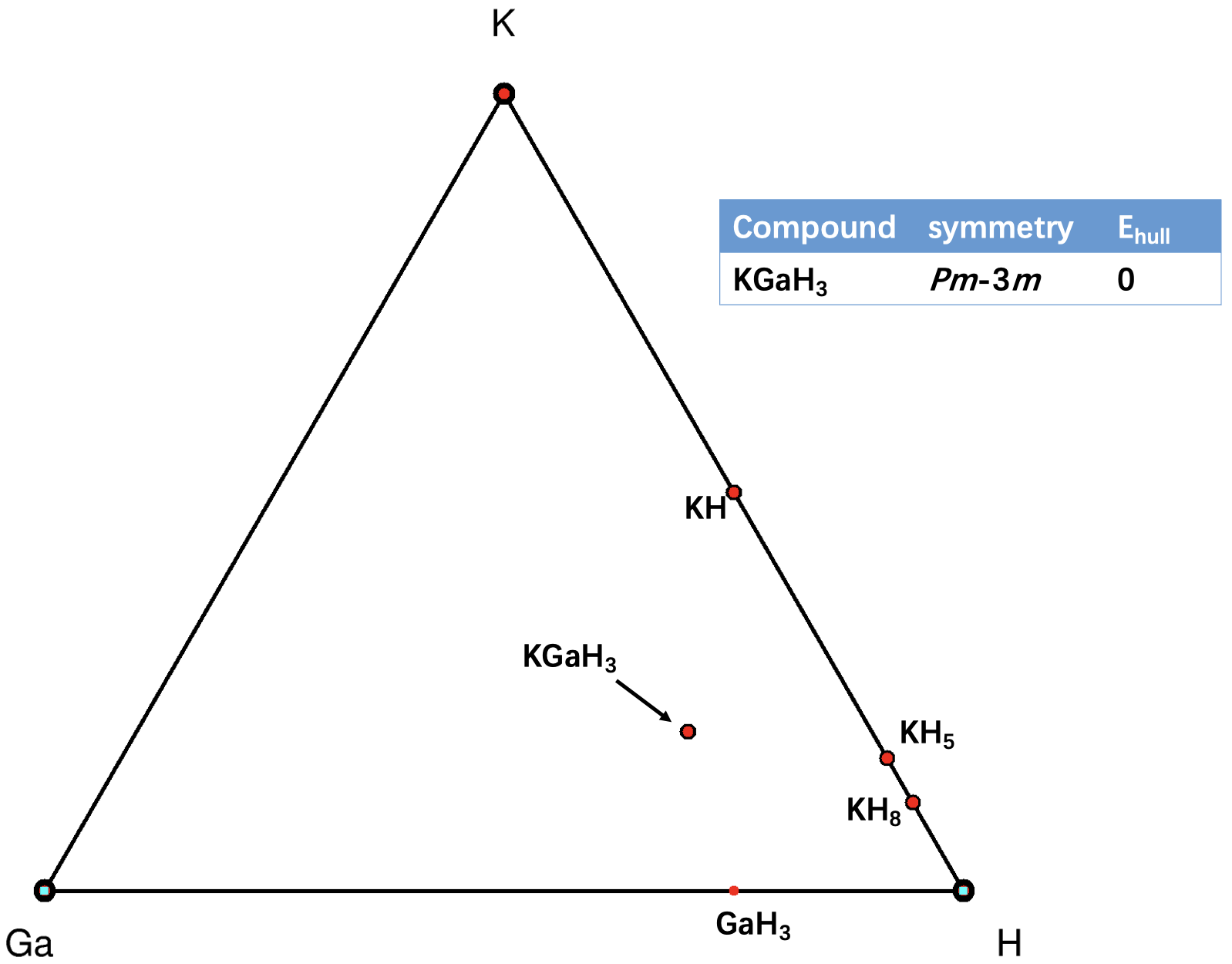


Figure S3 The convex hull of KGaH_3_ at 50 GPa. E_hull_ represent the distance to the convex hull of stability in meV per atom.


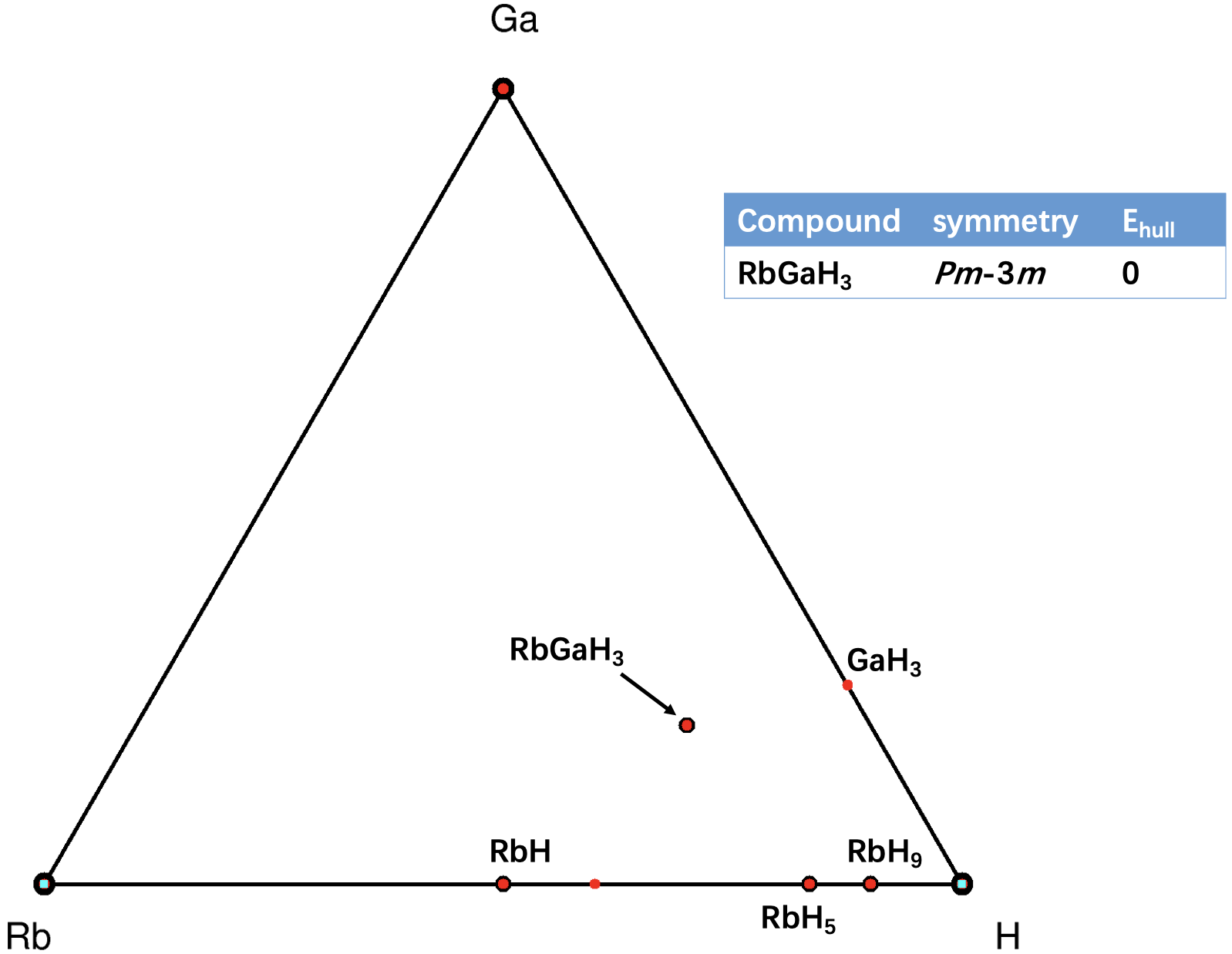


Figure S4 The convex hull of RbGaH_3_ at 50 GPa. E_hull_ represent the distance to the convex hull of stability in meV per atom.


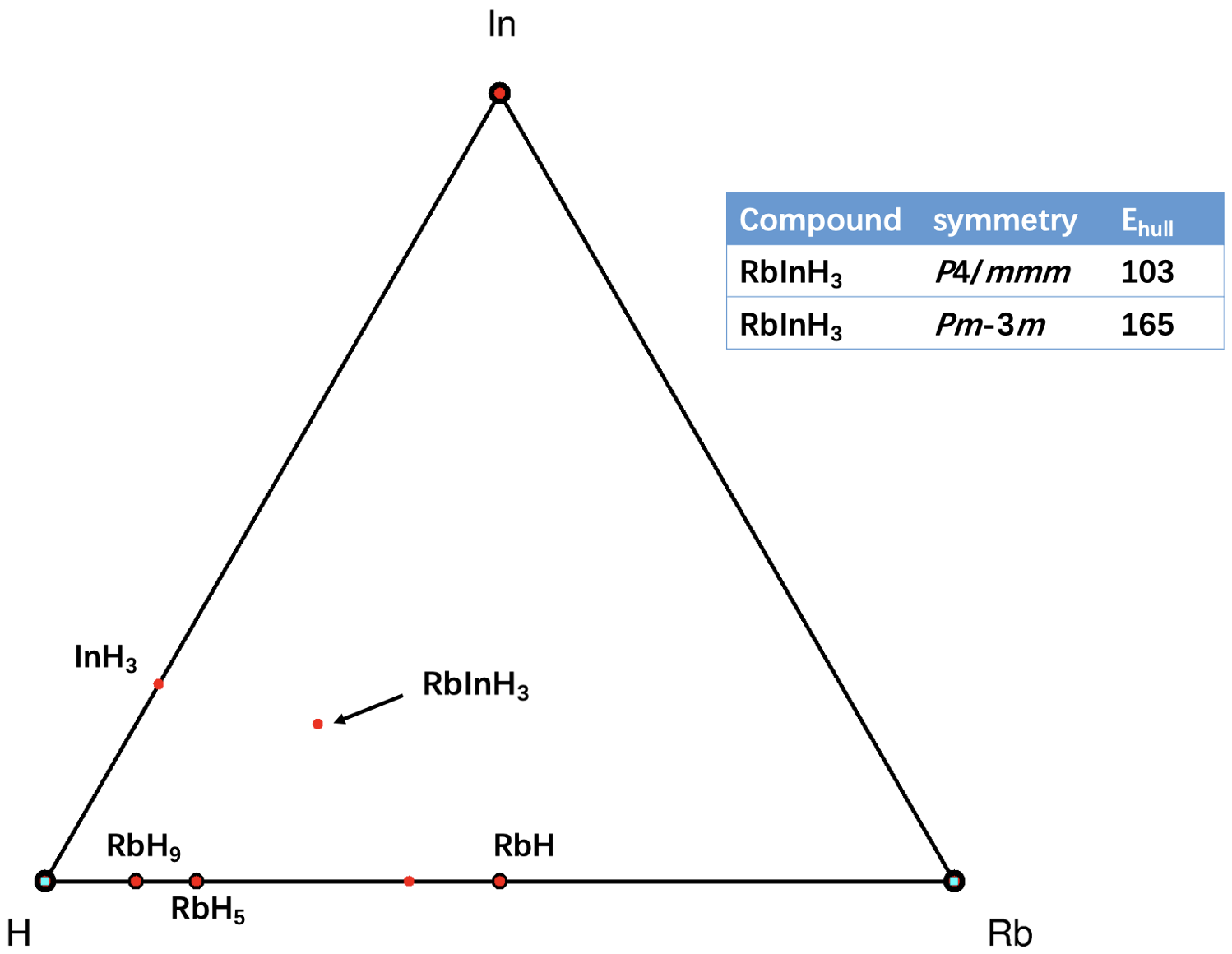


Figure S5 The convex hull of RbInH_3_ at 50 GPa. E_hull_ represent the distance to the convex hull of stability in meV per atom.


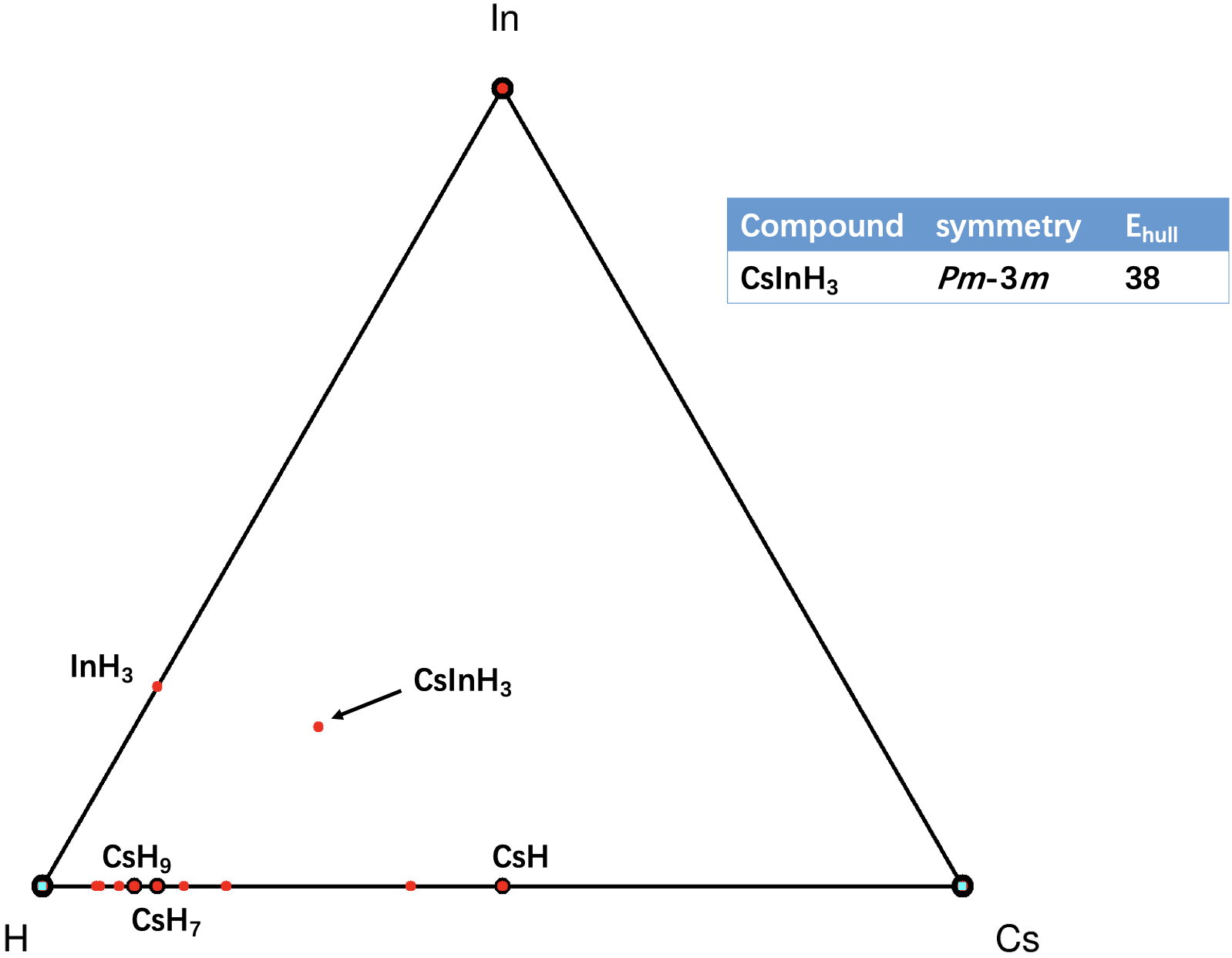


Figure S6 The convex hull of CsInH_3_ at 50 GPa. E_hull_ represent the distance to the convex hull of stability in meV per atom.


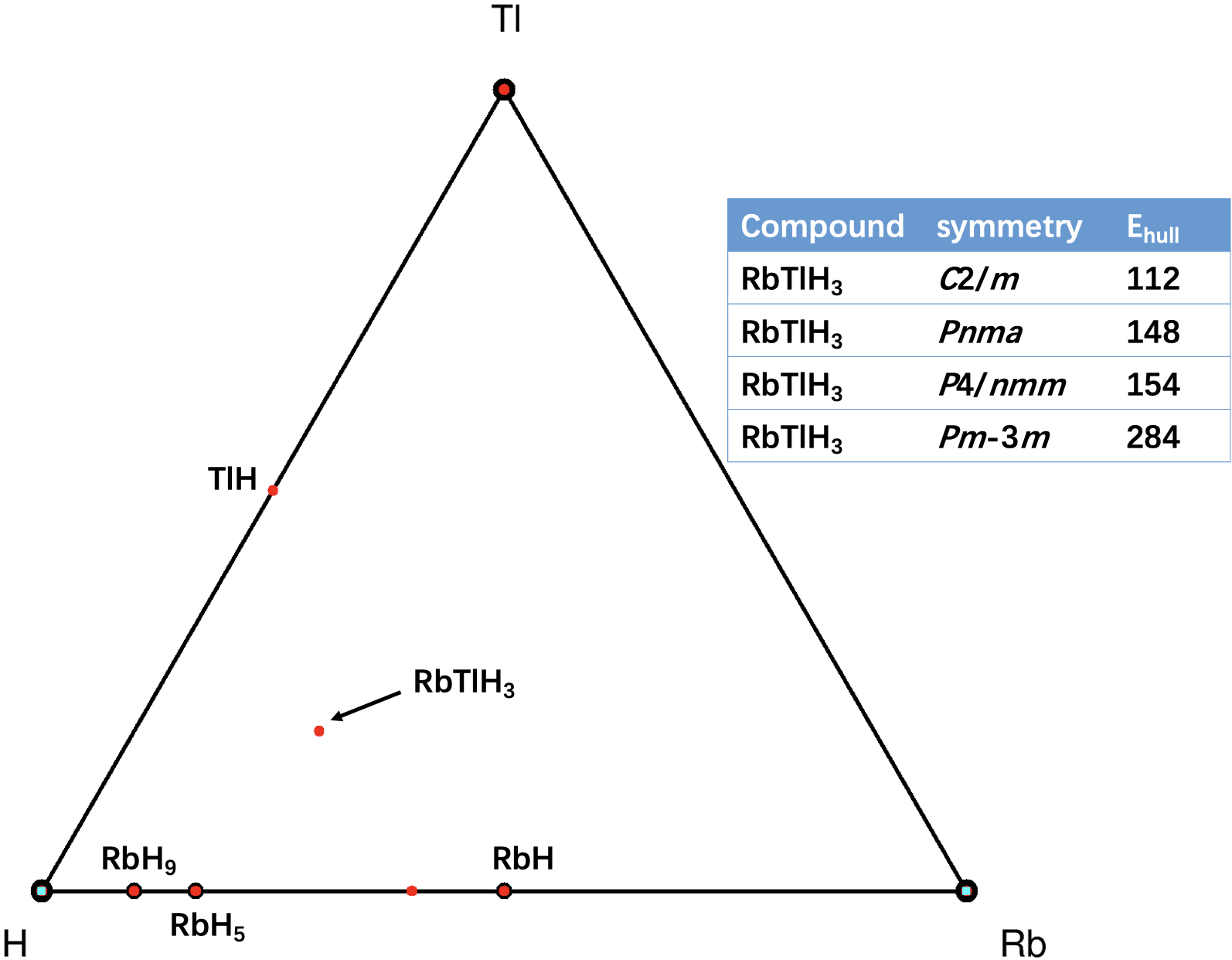


Figure S7 The convex hull of RbTlH_3_ at 50 GPa. E_hull_ represent the distance to the convex hull of stability in meV per atom.


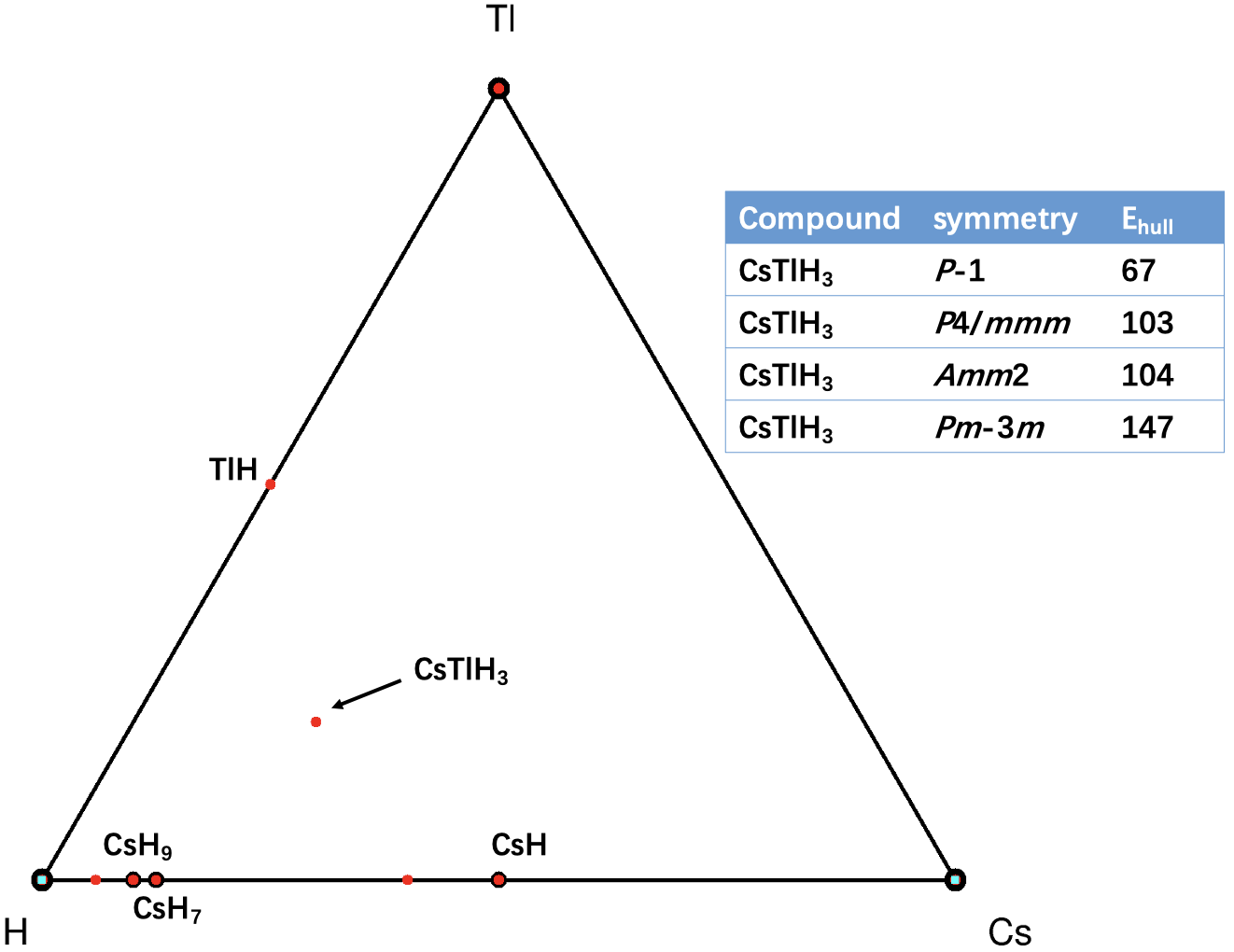


Figure S8 The convex hull of CsTlH_3_ at 50 GPa. E_hull_ represent the distance to the convex hull of stability in meV per atom.


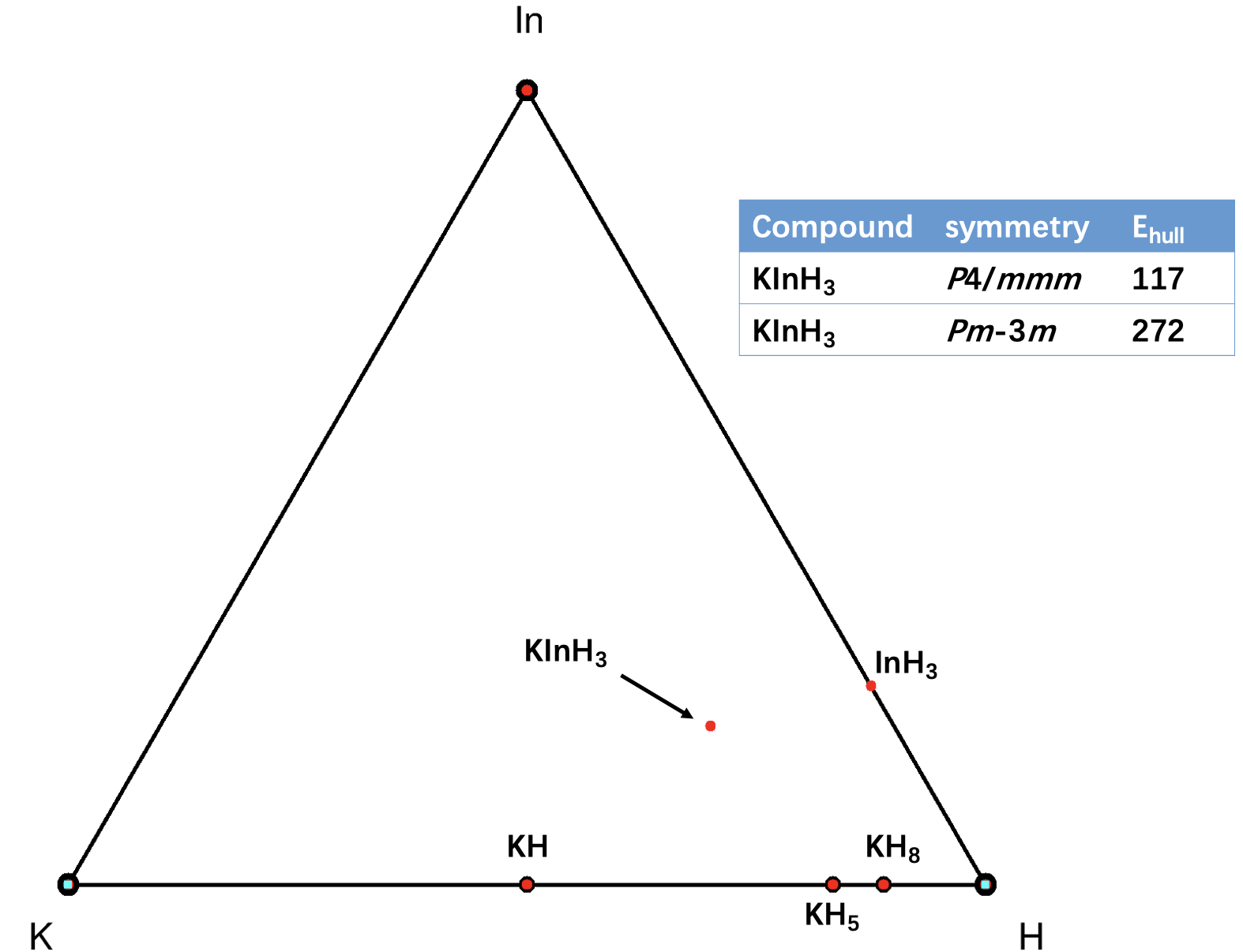


Figure S9 The convex hull of KInH_3_ at 50 GPa. E_hull_ represent the distance to the convex hull of stability in meV per atom.


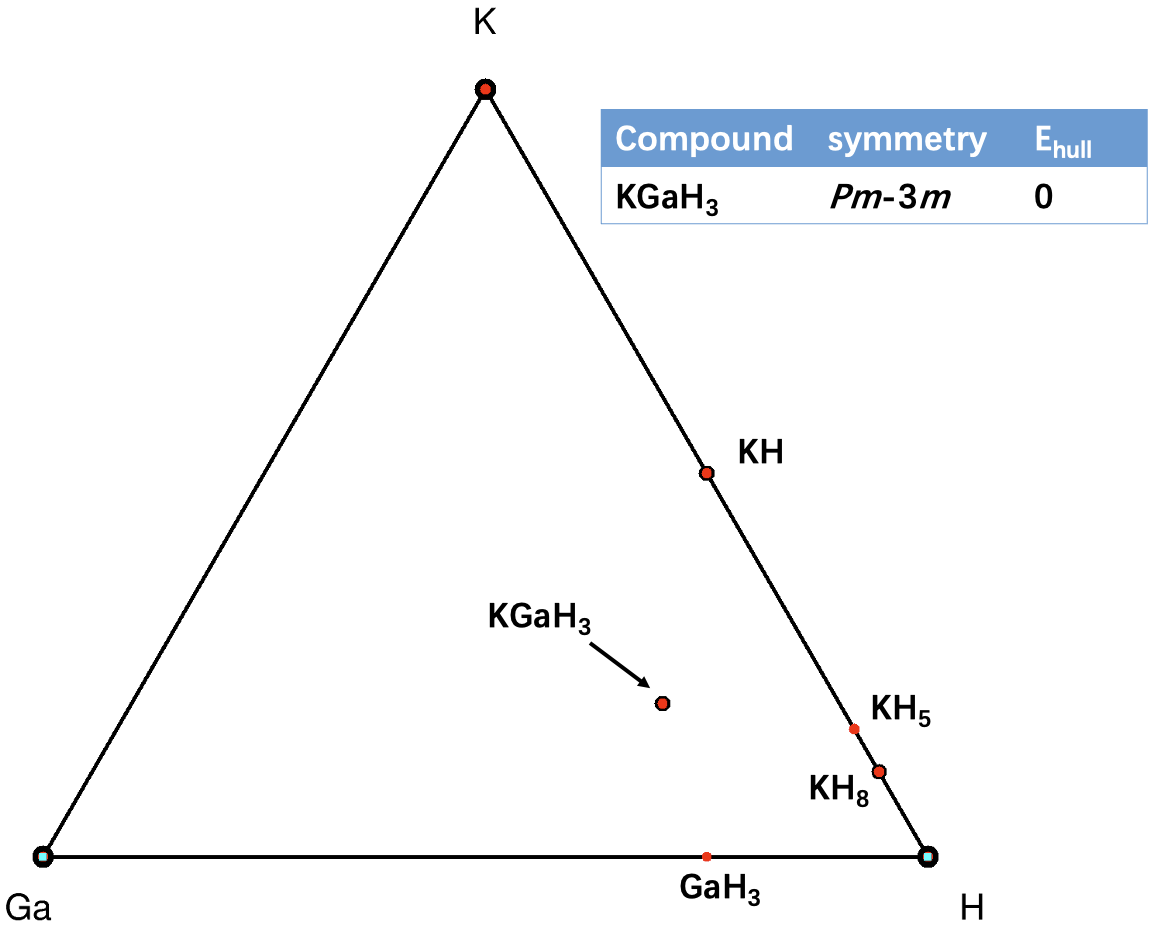


Figure S10 The convex hull of KGaH_3_ at 10 GPa. E_hull_ represent the distance to the convex hull of stability in meV per atom.


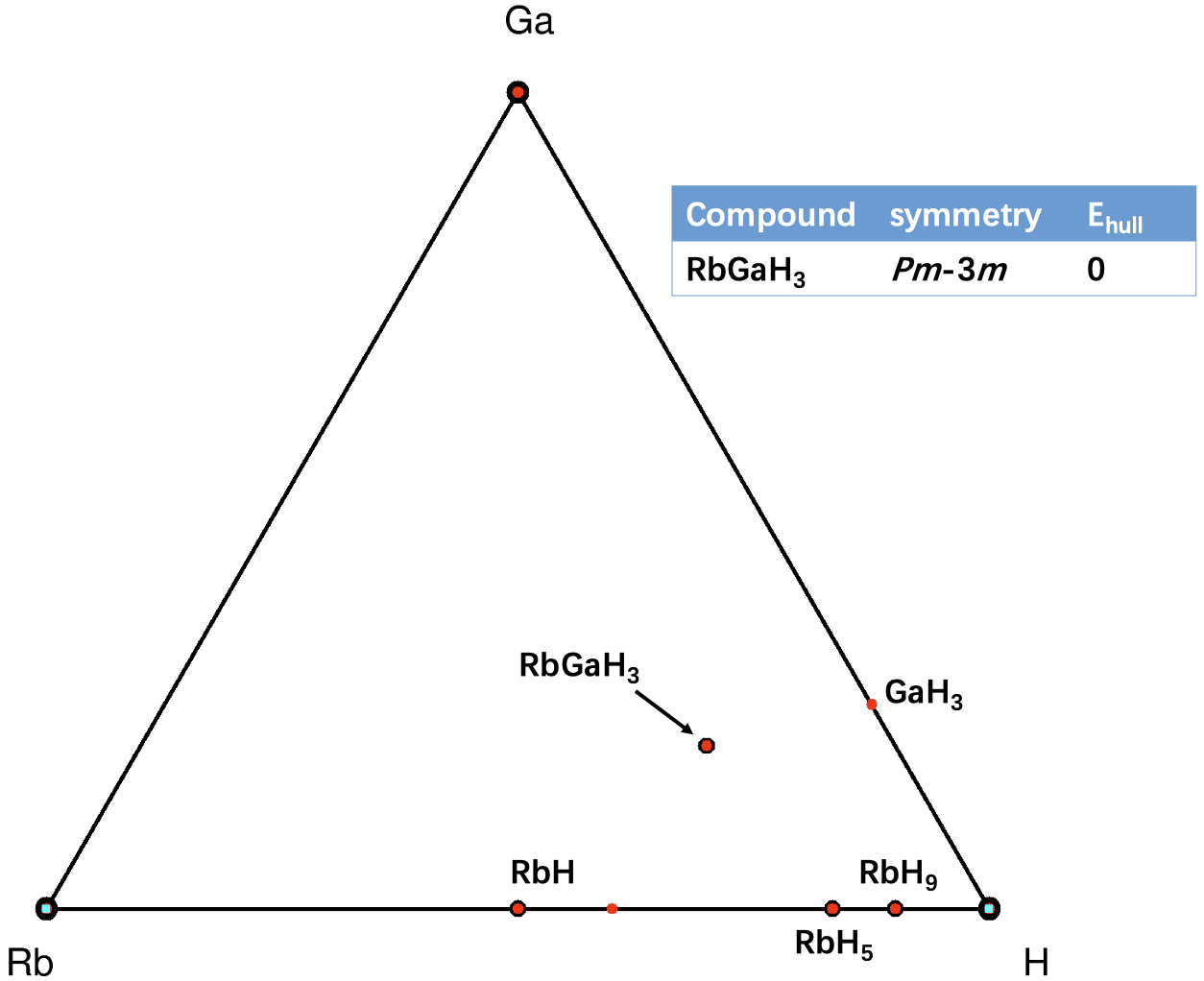


Figure S11 The convex hull of RbGaH_3_ at 20 GPa. E_hull_ represent the distance to the convex hull of stability in meV per atom.


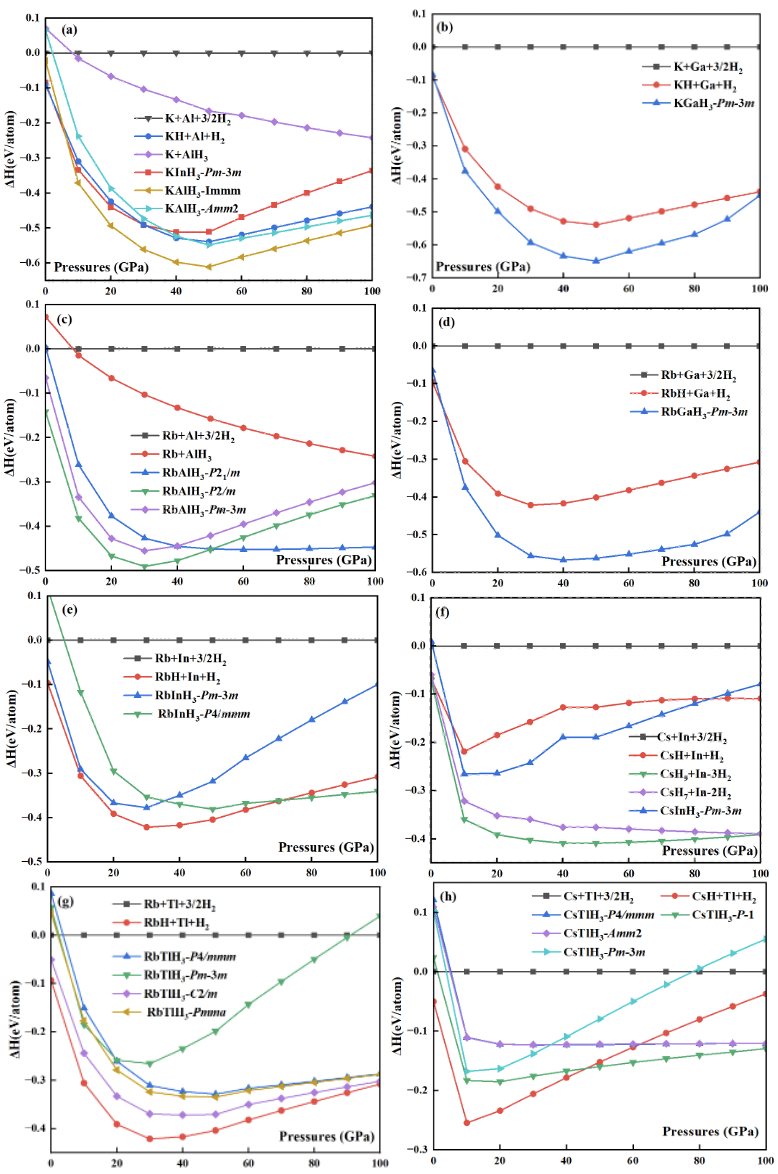


Figure S12 The enthalpy difference of AXH_3_ relative to elemental substances as a function of pressure.


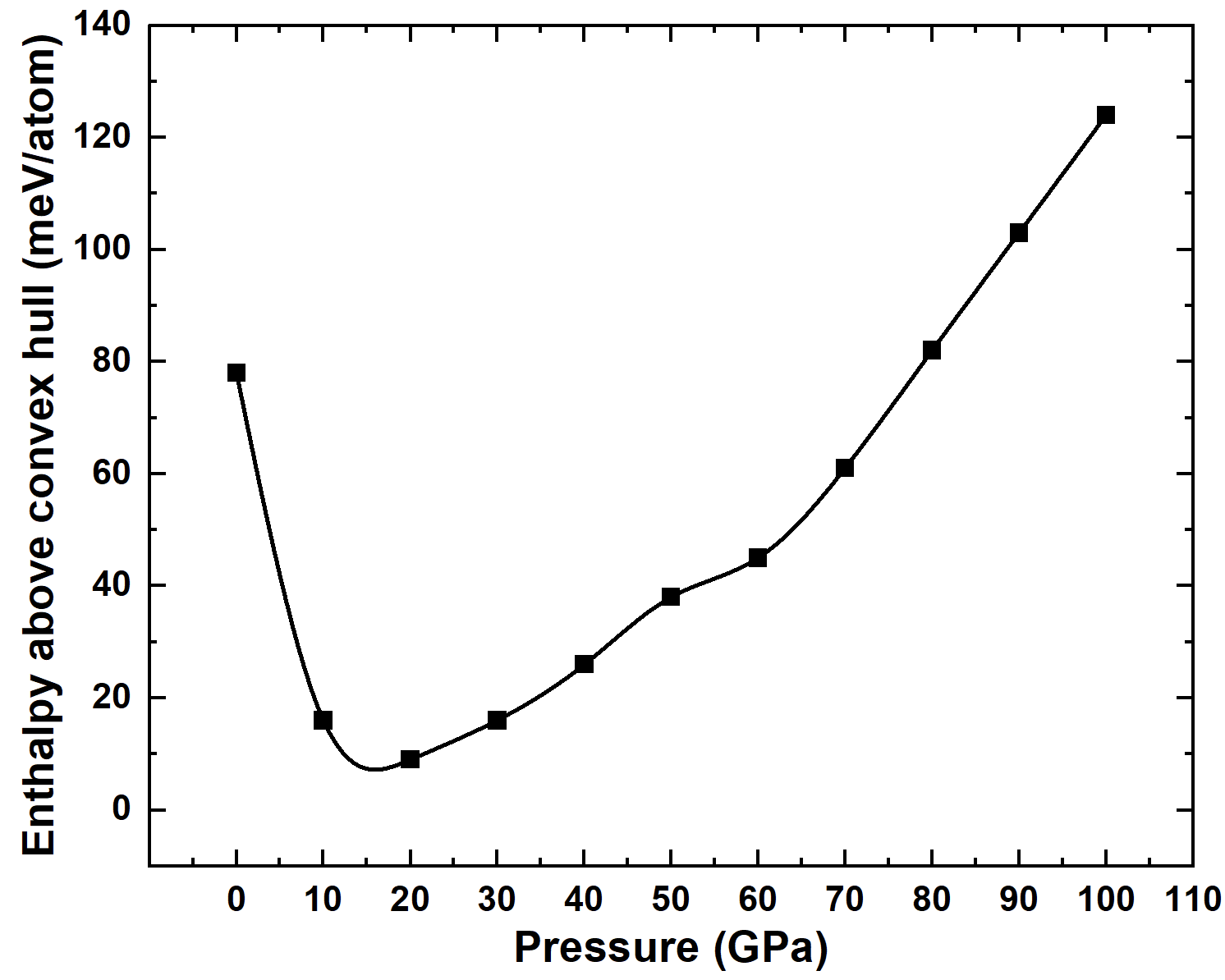


Figure S13 The enthalpy above convex hull of CsInH_3_ as a function of pressure.


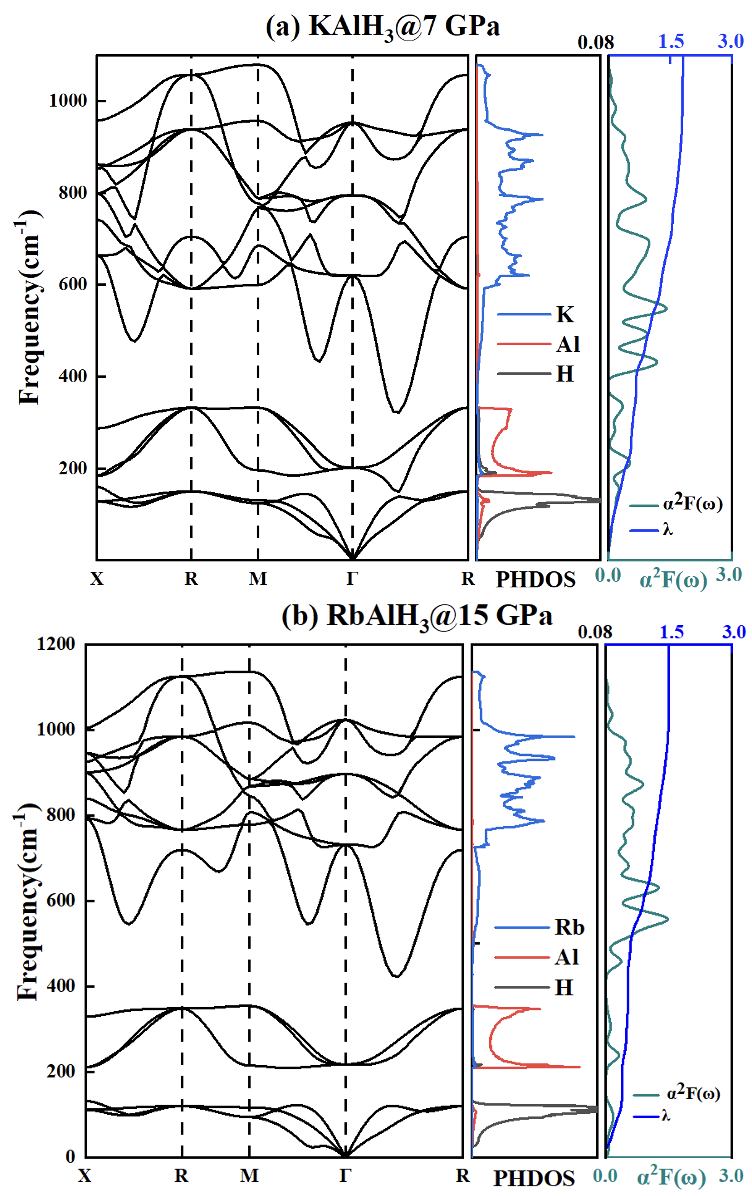


Figure S14 The phonon band structure and phonon density of states of (a) KAlH_3_ at 7 GPa and (b) RbAlH_3_ at 15 GPa.


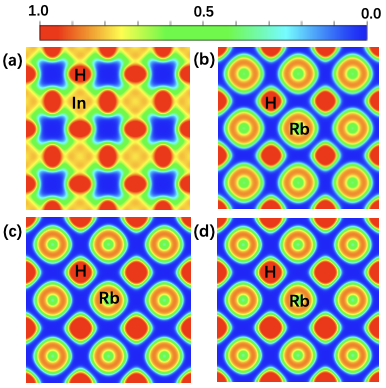


Figure S15 The 2-dimensional electron localization function (ELF) for perovskite hydrides (a) RbInH_3_ at IIIA group atomic layer and (b) RbGaH_3_, (c) RbInH_3_, (d) RbTlH_3_ at alkali metal layer.


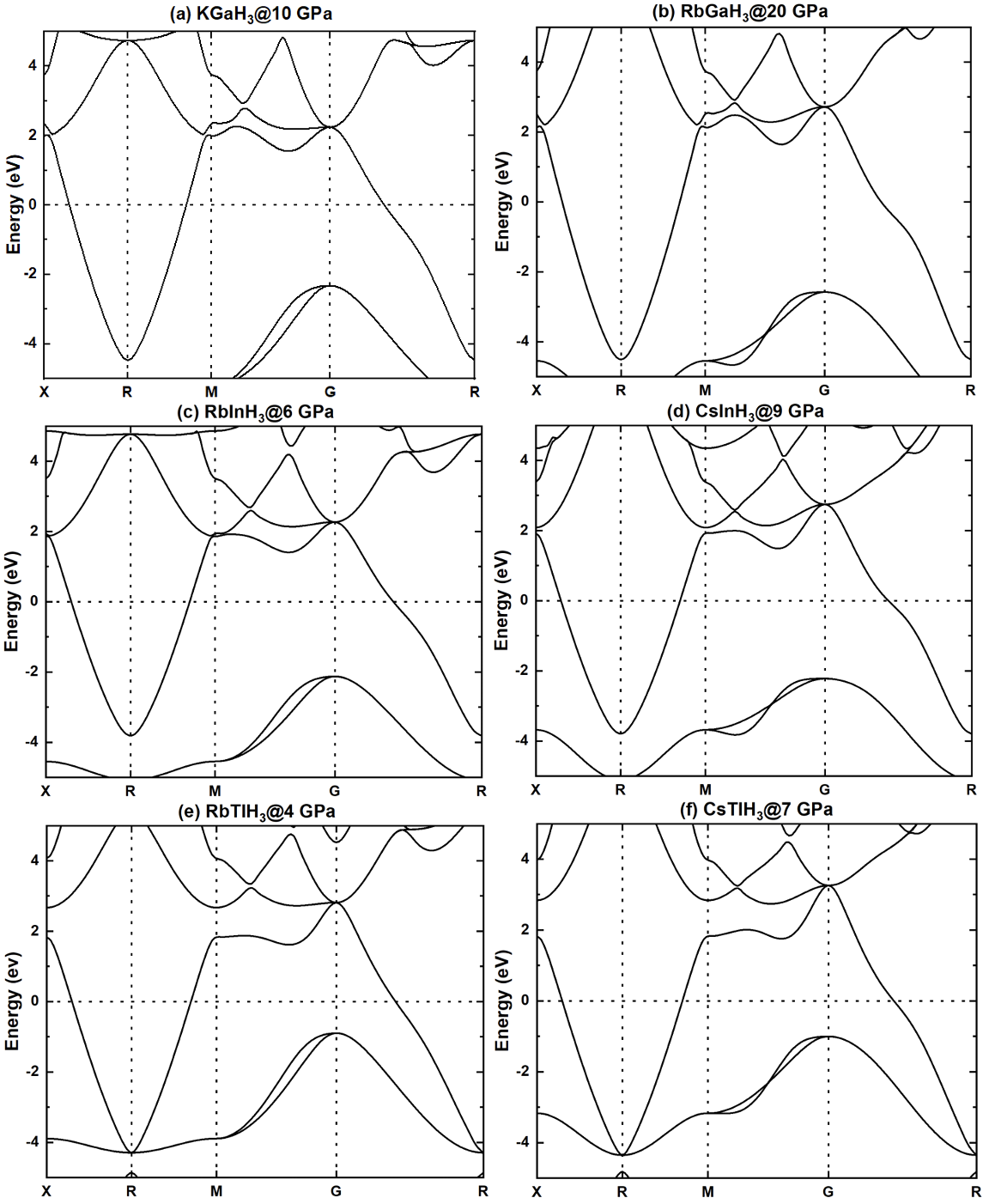


Figure S16 Calculated electronic band structures for (a) KGaH_3_ at 10 GPa, (b) RbGaH_3_ at 20 GPa, (c) RbInH_3_ at 6 GPa, (b) CsInH_3_ at 9 GPa, (e) RbTlH_3_ at 4 GPa and (f) CsTlH_3_ at 7 GPa.


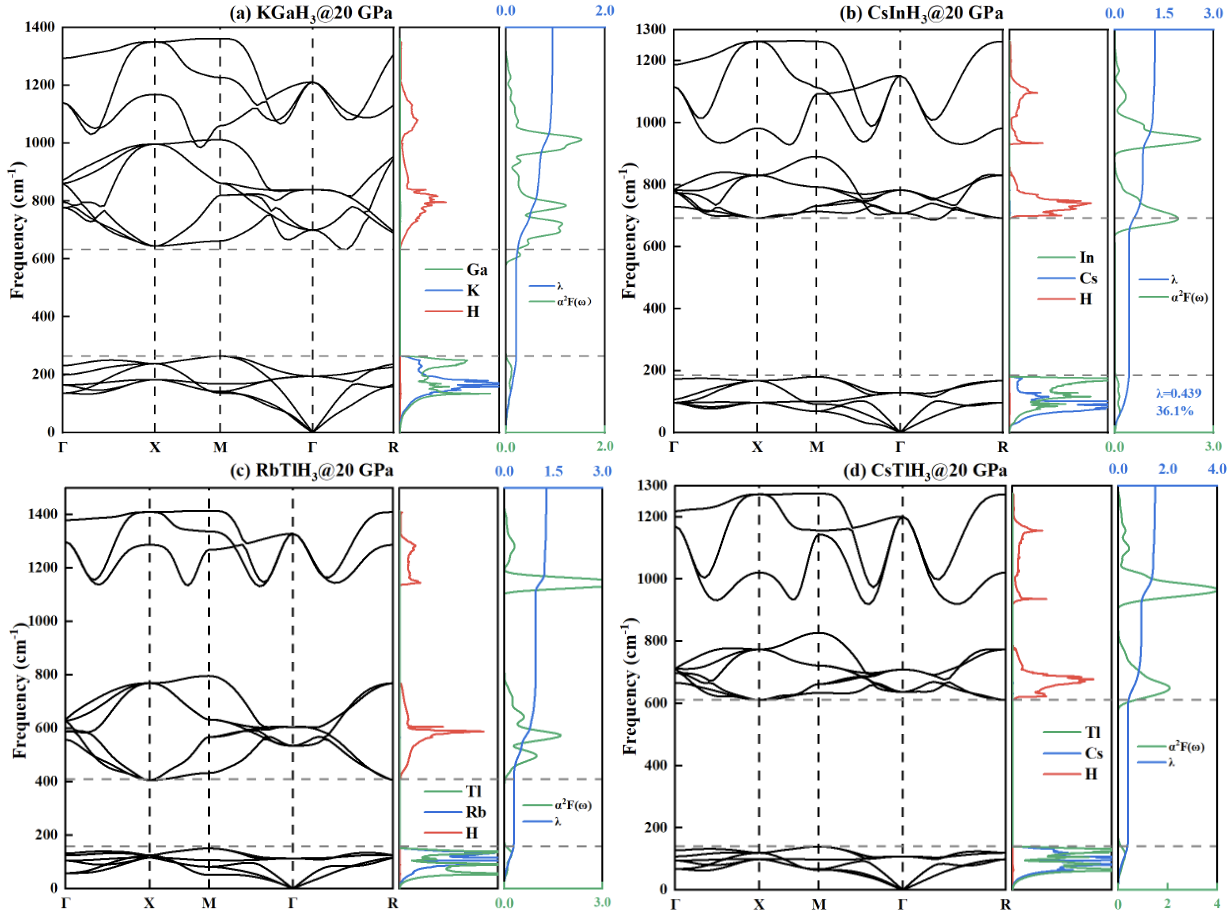


Figure S17 The phonon band structure and phonon density of states of (a) KGaH_3_, (b) CsInH_3_, (a) RbTlH_3_ and (b) CsTlH_3_ at 20 GPa.


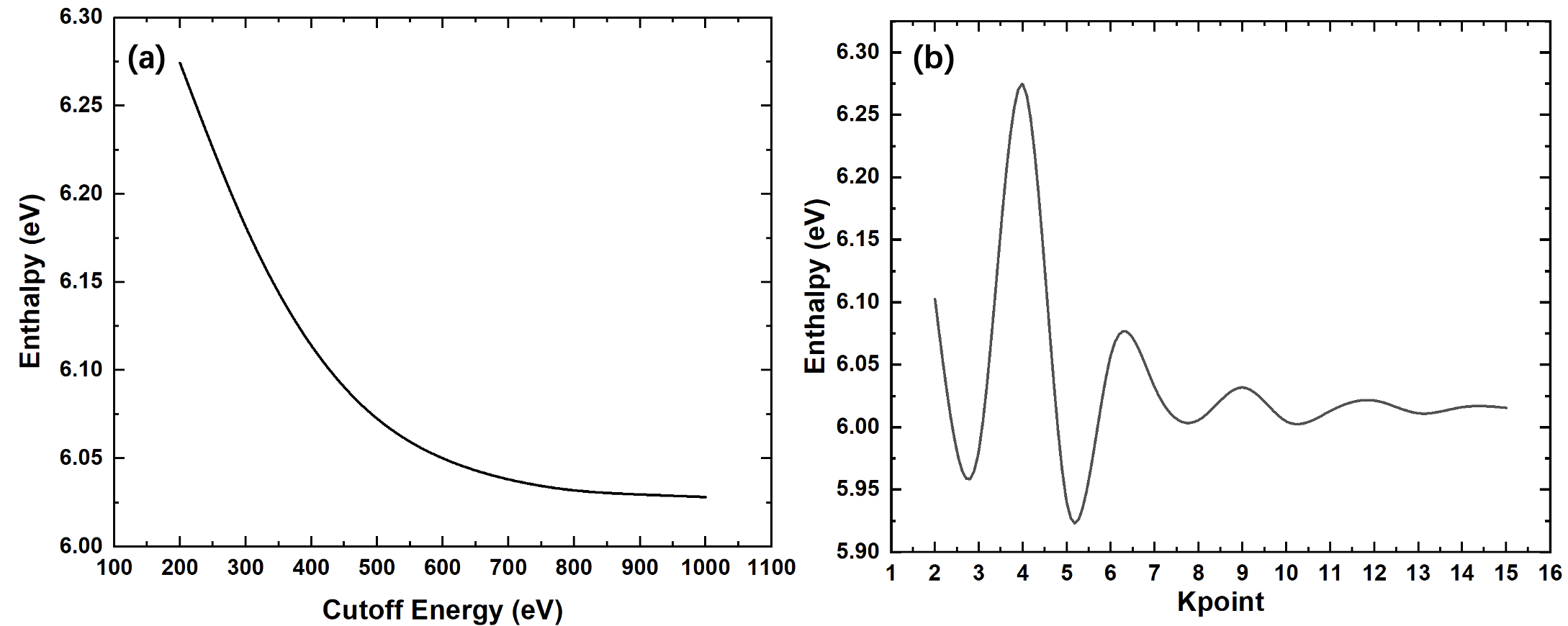


Figure S18 The enthalpy of CsInH_3_ as a function of (a)cut off energy and (b) K-point grid density using VASP.


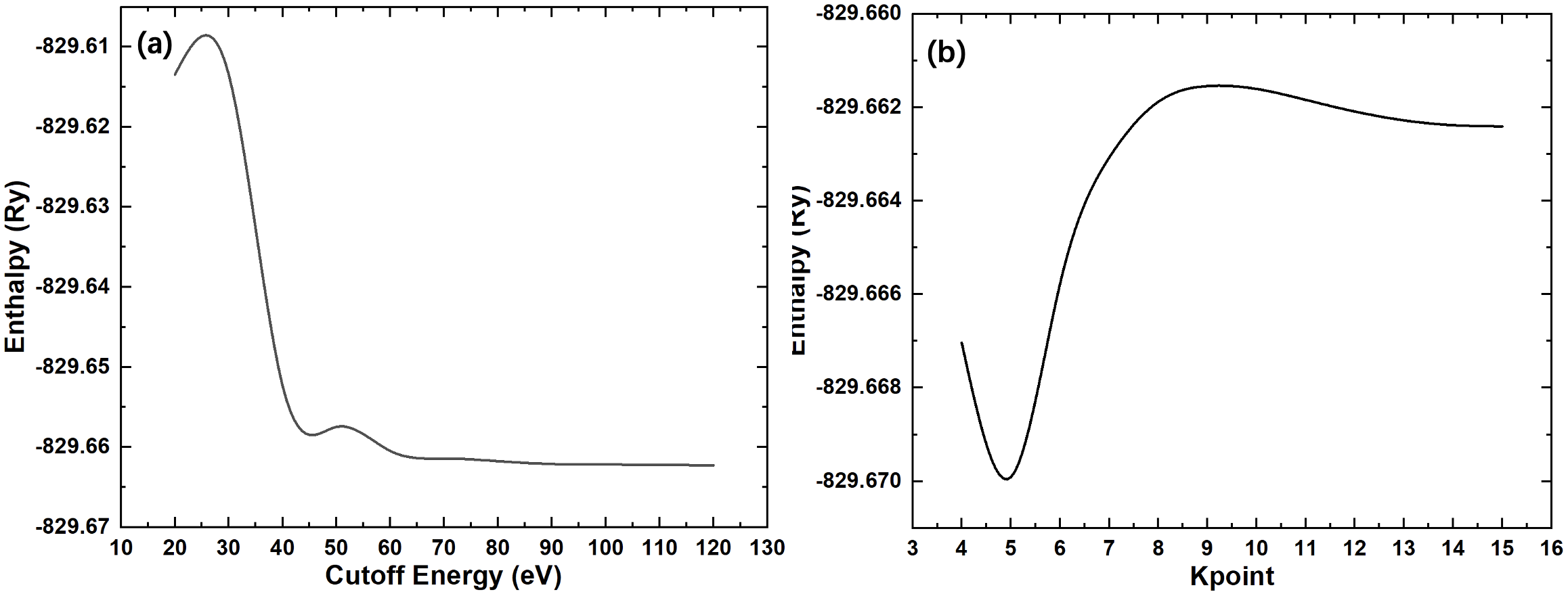


Figure S19 The enthalpy of CsInH_3_ as a function of (a)cut off energy and (b) K-point grid density using QE.


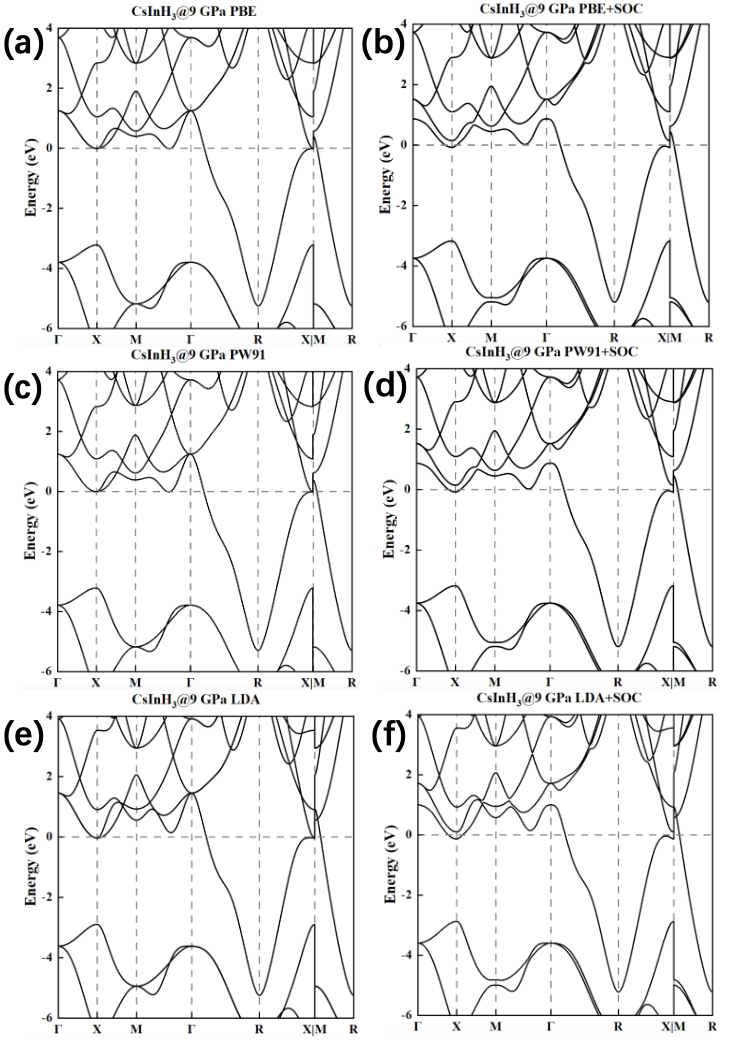


Figure S20 Calculated electronic band structures CsInH_3_ using (a) GGA-PBE without SOC, (b) GGA-PBE with SOC, (c) GGA-PW91 without SOC, (b) GGA-PW91 with SOC, (e) LDA without SOC and (f) LDA with SOC.


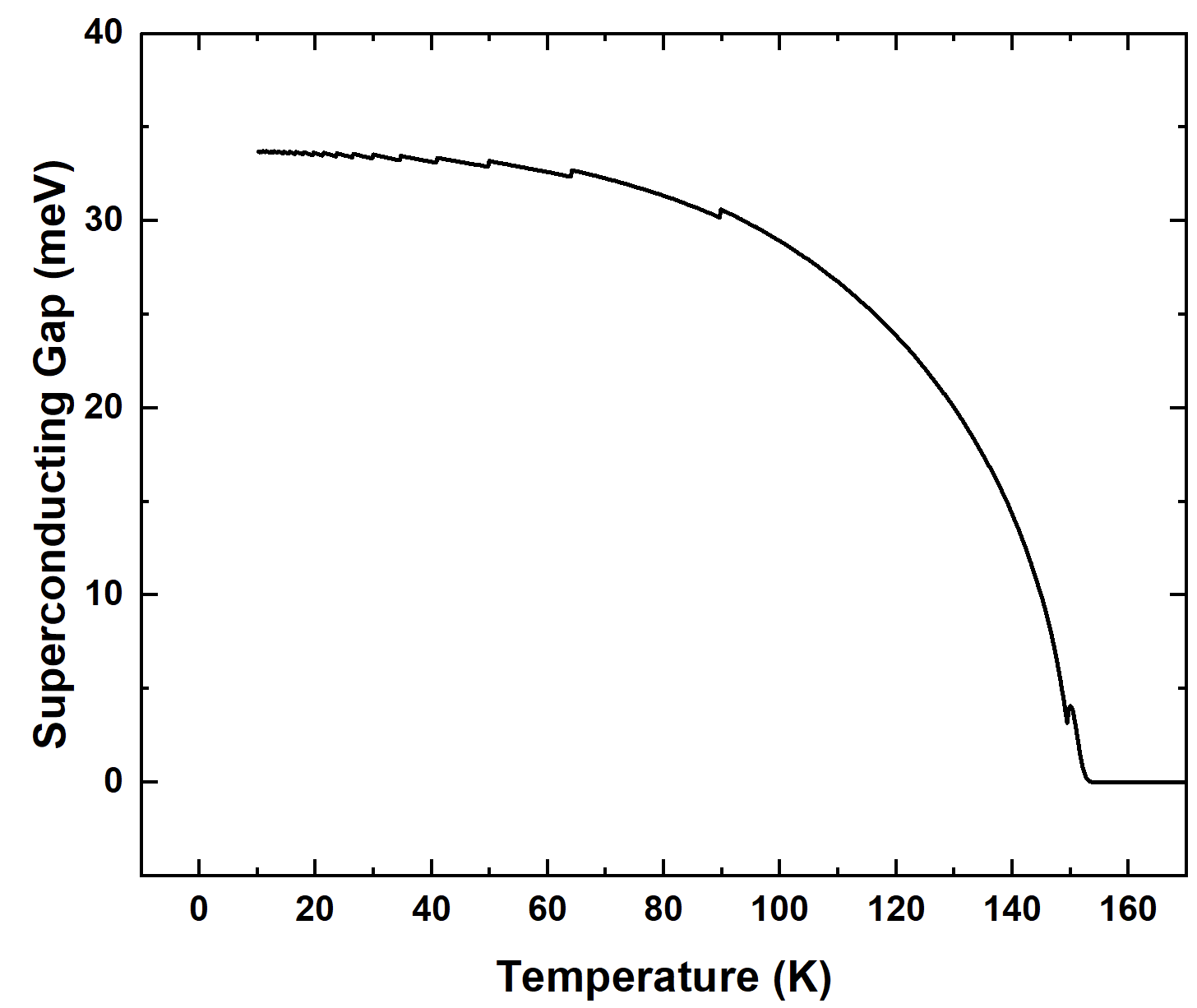


Figure S21 The superconducting gap of CsInH_3_ as a function of temperature obtained by scE equation.


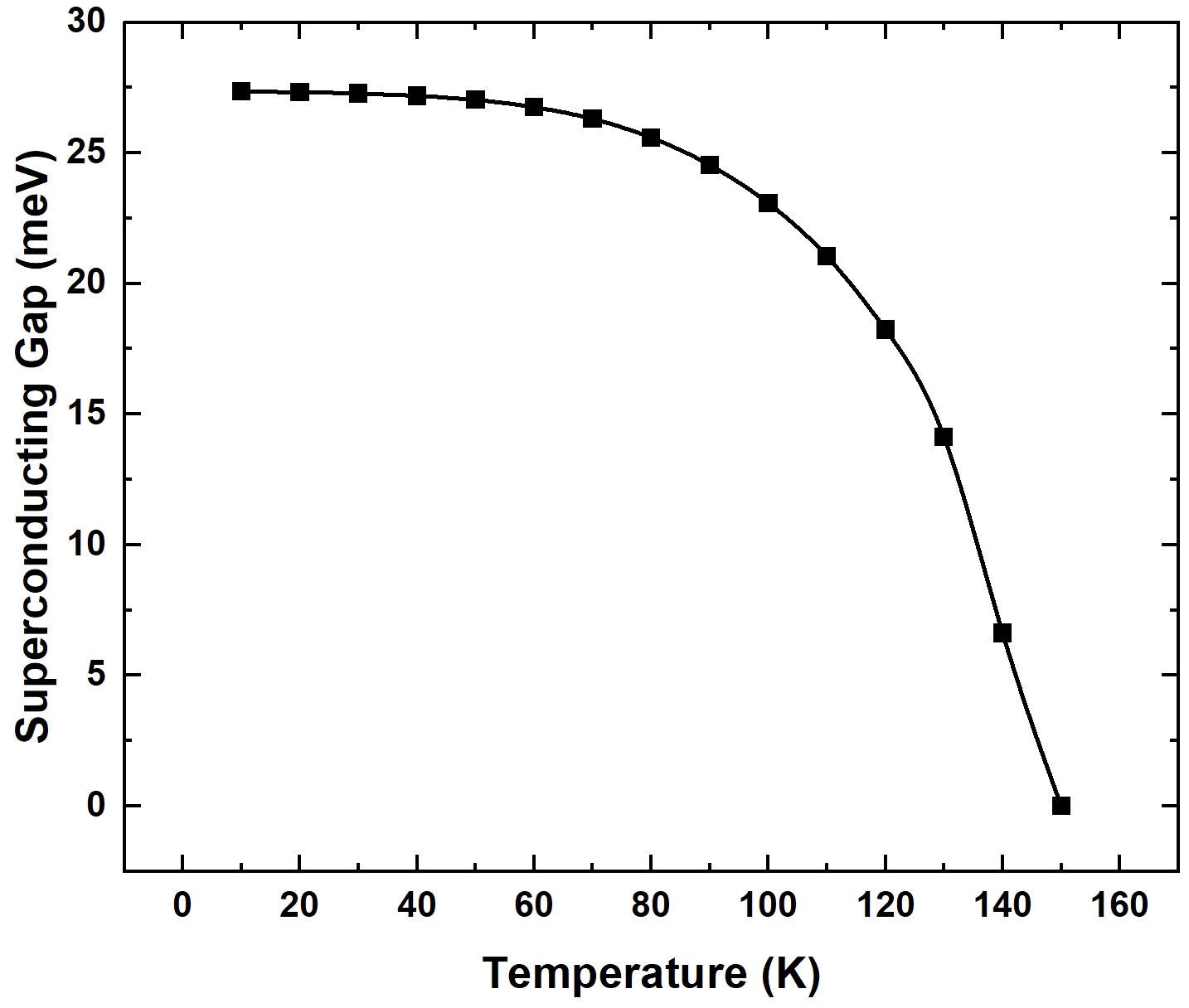


Figure S22 The isotropic superconducting gap of CsInH_3_ as a function of temperature obtained by EPW code.

# TABLES

Table S1 Lattice parameters and atomic positions of AXH_3_.

| **Structure** | **Parameters**  (Å, deg) | **Atom** | **x** | **y** | **z** |
| --- | --- | --- | --- | --- | --- |
| *Pm-3m*  KGaH_3_  (10 GPa) | a=b=c= 3.74787  α=β=γ= 90 | H  H  H  K  Ga | 0.5  0.5  0.0  0.0  0.5 | 0.0  0.5  0.5  0.0  0.5 | 0.5  0.0  0.5  0.0  0.5 |
| *Pm-3m*  RbGaH_3_  (20 GPa) | a=b=c= 3.69336  α=β=γ= 90 | H  H  H  Rb  Ga | 0.5  0.5  0.0  0.0  0.5 | 0.0  0.5  0.5  0.0  0.5 | 0.5  0.0  0.5  0.0  0.5 |
| *Pm-3m*  RbInH_3_  (6 GPa) | a=b=c= 4.13275  α=β=γ= 90 | H  H  H  Rb  In | 0.5  0.5  0.0  0.0  0.5 | 0.0  0.5  0.5  0.0  0.5 | 0.5  0.0  0.5  0.0  0.5 |
| *Pm-3m*  CsInH_3_  (9 GPa) | a=b=c= 4.14653  α=β=γ= 90 | H  H  H  Cs  In | 0.5  0.5  0.0  0.0  0.5 | 0.0  0.5  0.5  0.0  0.5 | 0.5  0.0  0.5  0.0  0.5 |
| *Pm-3m*  RbTlH_3_  (4 GPa) | a=b=c= 4.24476  α=β=γ= 90 | H  H  H  Rb  Tl | 0.5  0.5  0.0  0.0  0.5 | 0.0  0.5  0.5  0.0  0.5 | 0.5  0.0  0.5  0.0  0.5 |
| *Pm-3m*  CsTlH_3_  (7 GPa) | a=b=c= 4.24527  α=β=γ= 90 | H  H  H  Cs  Tl | 0.5  0.5  0.0  0.0  0.5 | 0.0  0.5  0.5  0.0  0.5 | 0.5  0.0  0.5  0.0  0.5 |

Table S2. The calculated elastic constants C_ij_ (GPa) of AXH_3_.

| Compound | C_11_ | C_12_ | C_44_ |
| --- | --- | --- | --- |
| KAlH_3_ | 115.61 | 57.46 | 25.39 |
| RbAlH_3_ | 152.93 | 85.81 | 58.80 |
| KGaH_3_ | 92.75 | 73.30 | 34.50 |
| RbGaH_3_ | 188.69 | 126.60 | 82.58 |
| RbInH_3_ | 127.22 | 45.76 | 27.21 |
| CsInH_3_ | 128.30 | 44.87 | 42.88 |
| RbTlH_3_ | 124.95 | 53.04 | 23.52 |
| CsTlH_3_ | 121.98 | 52.03 | 36.71 |

Table S3. The calculated electron-phonon coupling (EPC) parameter λ, logarithmic average phonon frequency ω_log_, superconducting critical temperature f_1_f_2_*T_c_* using Allen-Dynes modified McMillan equation and *T_c_*^scE^ using the Self-consistent solution of the Eliashberg equation for AXH_3_ found in this work. The Coulomb pseudopotential μ^∗^ = 0.10 and 0.13.

| Compound | q-points meshes | Pressure  (GPa) | λ | ω_log_ (K) | f_1_f_2_T_c_ (K) | T_c_ ^scE^ (K) |
| --- | --- | --- | --- | --- | --- | --- |
| KAlH_3_ | 6×6×6 | 7 | 1.80 | 529 | 65-70 | 87-96 |
| RbAlH_3_ | 6×6×6 | 15 | 1.54 | 388 | 42-45 | 78-86 |
| KGaH_3_ | 6×6×6 | 10 | 2.72 | 585 | 93-99 | 138-146 |
| KGaH_3_ | 6×6×6 | 15 | 1.27 | 812 | 69-78 | 88-98 |
| KGaH_3_ | 6×6×6 | 20 | 0.94 | 827 | 44-52 | 52-61 |
| RbGaH_3_ | 6×6×6 | 20 | 1.76 | 715 | 84-91 | 117-127 |
| RbInH_3_ | 6×6×6 | 6 | 2.08 | 639 | 88-94 | 121-130 |
| CsInH_3_ | 6×6×6 | 9 | 2.92 | 546 | 91-96 | 144-153 |
| CsInH_3_ | 6×6×6 | 10 | 2.37 | 584 | 88-93 | 135-144 |
| CsInH_3_ | 6×6×6 | 15 | 1.49 | 583 | 60-66 | 92-102 |
| CsInH_3_ | 6×6×6 | 20 | 1.24 | 465 | 39-43 | 60-69 |
| RbTlH_3_ | 6×6×6 | 4 | 4.27 | 395 | 77-80 | 162-170 |
| RbTlH_3_ | 6×6×6 | 5 | 3.22 | 477 | 84-88 | 155-164 |
| RbTlH_3_ | 6×6×6 | 10 | 1.92 | 632 | 82-88 | 122-132 |
| RbTlH_3_ | 6×6×6 | 15 | 1.50 | 670 | 70-76 | 98-108 |
| RbTlH_3_ | 6×6×6 | 20 | 1.28 | 664 | 57-64 | 71-81 |
| CsTlH_3_ | 6×6×6 | 7 | 3.69 | 478 | 89-93 | 156-163 |
| CsTlH_3_ | 6×6×6 | 10 | 2.35 | 599 | 90-95 | 139-148 |
| CsTlH_3_ | 6×6×6 | 15 | 1.74 | 665 | 80-86 | 116-128 |
| CsTlH_3_ | 6×6×6 | 20 | 1.48 | 669 | 69-75 | 99-110 |

# Equations for calculating *T*_c_ and related parameters

**(1) The Allen−Dynes-modified McMillan equation**

*T*_c_ can be estimated by the McMillan equation[1]:

$T_{c}=\frac{\omega_{log}}{1.2}exp\left[ -\frac{1.04\left( 1+\lambda\right)}{\lambda-\mu^{*}\left( 1+0.62\lambda\right)} \right]$ (1)

where λ and ω_log_ are the electron-phonon coupling constant and the logarithmic-averaged phonon frequency, respectively, and μ^*^ is the Coulomb pseudopotential, for which we use the widely accepted range of 0.1-0.13. λ and ω_log_ are given by

$\lambda=2\int_{0}^{\infty} \frac{\alpha^{2}F(\omega)}{\omega}d\omega$ (2)

and

$\omega_{log}=exp\left( \frac{2}{\lambda}\int_{0}^{\infty} \frac{d\omega}{\omega}\alpha^{2}F(\omega)\ln\omega\right)$ (3)

The parameter ω denotes the phonon frequency, and α^2^F(ω) is the Eliashberg spectral function

$\alpha^{2}F\left( \omega\right)=\frac{1}{2\pi N(\varepsilon_{F})}\sum_{\boldsymbol{q}\upsilon} \frac{\gamma_{\boldsymbol{q}\upsilon}}{\omega_{\boldsymbol{q}\upsilon}}\delta(\omega-\omega_{\boldsymbol{q}\upsilon})$ (4)

The line width γ_q,υ_ is written as

$\gamma_{q\upsilon}=\pi\omega_{q\upsilon}\sum_{mn} \sum_{k} \left| g_{mn}^{\nu}\left( \boldsymbol{k},\boldsymbol{q} \right) \right|^{2}\delta(\varepsilon_{m,\boldsymbol{k}+\boldsymbol{q}}-\varepsilon_{F})\times\delta(\varepsilon_{n,\boldsymbol{k}}-\varepsilon_{F})$ (5)

where ε_n,_**_k_** is the energy of the bare electronic Bloch state, ε_F_ is the Fermi energy, and $g_{mn}^{\nu}\left( \boldsymbol{k},\boldsymbol{q} \right)$ is the electron−phonon matrix element.

When the value of $\lambda$ larger than 1.3, strong-coupling corrections begin to appear. Therefore, P. B. Allen and R. C. Dynes use two separate correction factors ($f_{1}$ and $f_{2}$) to describe these two effects. Then we can further obtain the Allen−Dynes-modified McMillan equation[2]:

$T_{c}=\frac{f_{1}f_{2}\omega_{log}}{1.2}exp\left[ -\frac{1.04\left( 1+\lambda\right)}{\lambda-\mu^{*}\left( 1+0.62\lambda\right)} \right]$ (6)

$f_{1}$ and $f_{2}$ are given by

$f_{1}=\sqrt[3]{\left[ 1+\left( \frac{\lambda}{2.46(1+3.8\mu^{*})} \right)^{\frac{3}{2}} \right]}$ (7)

and

$f_{2}=1+\frac{\left( \frac{\omega_{2}}{\omega_{log}}-1 \right)\lambda^{2}}{\lambda^{2}+\left[ 1.82(1+6.3\mu^{*})\frac{\bar{\omega}_{2}}{\omega_{log}} \right]}$ (8)

average frequencies $\bar{\omega}_{2}$ is given by

$\bar{\omega}_{2}=\sqrt{\frac{2}{\lambda}}\int_{0}^{\infty} \frac{d\omega}{\omega}\alpha^{2}F(\omega)\omega d\omega$ (9)

**(2) Self-consistent solution of the Eliashberg equation**

For strong-coupling system, it can be better described with Eliashberg equation[3]:

$Z\left( i\omega_{n} \right)\Delta\left( i\omega_{n} \right)=\frac{\pi T}{N_{F}}\sum_{n^{'}} \frac{\Delta\left( i\omega_{n}^{'} \right)}{\sqrt{\omega_{n}^{'2}+\Delta^{2}\left( i\omega_{n}^{'} \right)}}\times[\lambda(\omega_{n}-\omega_{n^{'}})-N_{F}\mu^{*}]\delta(\epsilon)$ (10)

$\begin{aligned} Z(i\omega_{n})=1+\frac{\pi T}{N_{F}\omega_{n}}\sum_{n^{'}} \frac{\omega_{n}^{'}}{\sqrt{\omega_{n}^{'2}+\Delta^{2}\left( i\omega_{n}^{'} \right)}}\lambda\left( \omega_{n}-\omega_{n^{'}} \right)\delta\left( \epsilon\right) \end{aligned}$ (11)

where functions $Z(i\omega_{n})$ and $\Delta\left( i\omega_{n} \right)$ are the renormalization function and pairing order parameter, respectively. $N_{F}$ is the density of electronic states at the Fermi level, and $\delta(\epsilon)$ is the Dirac delta function. ${i\omega}_{n}=i(2n+1)\pi T_{c}$ are the fermion Matsubara frequencies. $\mu^{*}$is the Coulomb pseudopotential, for which we use the widely accepted range of 0.1 - 0.13. $\lambda(\omega_{n}-\omega_{n^{'}})$ contains the electron-phonon coupling matrix, phonon propagator, and the phonon density of states, and is given by:

$\lambda(\omega_{n}-\omega_{n^{'}})=\int_{0}^{\infty} d\omega\frac{2\omega}{(\omega_{n}-\omega_{n}^{'})^{2}+\omega^{2}}\alpha^{2}F(\omega)$ (12)

The equations for the $Z(i\omega_{n})$ and $\Delta\left( i\omega_{n} \right)$ form a coupled nonlinear system and are solved self-consistently. We evaluated renormalization function and the order parameter for each Matsubara frequency along the imaginary energy axis. After calculating $Z(i\omega_{n})$ and $\Delta\left( i\omega_{n} \right)$, an analytic continuation is performed to the real axis using Pade’ functions.

**References**

[1] W. L. McMillan, *Phys. Rev.* **1968**, *167*, 331.

[2] P. B. Allen, R. C. Dynes, *Phys. Rev. B* **1975**, *12* (3), 905, https://doi.org/10.1103/PhysRevB.12.905.

[3] G. M. Eliashberg, *Sov Phys Jetp* **1960**, *11:3* (3), 696.
